# Human vaccine responses regulated by parallel cytokine pathways

**DOI:** 10.1038/s41590-026-02547-x

**Published:** 2026-06-12

**Authors:** Guangbo Chen, Jing Guo, John Heath, Tyler R. Prestwood, Woo Joo Kwon, Ashley R. Smith, Tran T. Nguyen, Karan R. Kathuria, Elsa Sola, AbuBakr Sangare, Vamsee Mallajosyula, Ryan Furuichi Fong, Azam Mohsin, Lei Chen, Oviya Siva, Cindy Padilla, Mingdian Tan, Cornelia L. Dekker, Philip Grant, Ying Lu, Harry B. Greenberg, William H. Robinson, Catherine Blish, Shai S. Shen-Orr, Ahmad Salehi, Holden T. Maecker, Purvesh Khatri, Paul J. Utz, Yueh-Hsiu Chien, Mark M. Davis

**Affiliations:** 1 Institute for Immunology, Transplantation and Infection (ITI), Stanford University School of Medicine, Palo Alto, CA USA; 2 Department of Obstetrics and Gynecology, Medical College of Wisconsin, Milwaukee, WI USA; 3 Center for Immunology, Medical College of Wisconsin, Milwaukee, WI USA; 4 Department of Microbiology and Immunology, Medical College of Wisconsin, Milwaukee, WI USA; 5 Hematopoiesis and Immunobiology Program, Versiti Blood Research Institute, Milwaukee, WI USA; 6 Department of Microbiology and Immunology, Stanford University School of Medicine, Palo Alto, CA USA; 7 Department of Bioengineering, Stanford University, Stanford, CA USA; 8 Department of Medicine, Stanford University School of Medicine, Palo Alto, CA USA; 9 The Human Immune Monitoring Center (HIMC), Stanford University School of Medicine, Palo Alto, CA USA; 10 Department of Biology, Stanford University, Stanford, CA USA; 11 Division of Infectious Diseases and Geographic Medicine, Department of Medicine, Stanford University School of Medicine, Palo Alto, CA USA; 12 Asian Liver Center, Department of Surgery, Stanford University School of Medicine, Palo Alto, CA USA; 13 Department of Pediatrics (Infectious Diseases), Stanford University School of Medicine, Palo Alto, CA USA; 14 Department of Biomedical Data Science, Stanford University School of Medicine, Palo Alto, CA USA; 15 Division of Gastroenterology and Hepatology, Department of Medicine, Stanford University School of Medicine, Palo Alto, CA USA; 16 Ruth and Bruce Rappaport Faculty of Medicine, Technion–Israel Institute of Technology, Haifa, Israel; 17 Donor Network West, San Ramon, CA USA; 18 Division of Computational Medicine, Department of Medicine, Stanford University School of Medicine, Palo Alto, CA USA; 19 Program in Immunology, Stanford University School of Medicine, Palo Alto, CA USA

**Keywords:** Cytokines, Biotechnology, Influenza virus, Adjuvants

## Abstract

Human vaccine responses vary widely, but the determinants remain incompletely defined. Here we analyzed 66 cytokines across four inactivated influenza vaccine (IIV) cohorts over five seasons (*n* = 581) and identified baseline serum interleukin (IL)-18 and interferon (IFN)-β as correlates of day 28 antibody responses. To test causality, we evaluated 19 cytokines in human tonsil and spleen organoids and found that type I IFNs, IL-21 and IL-12, but not IL-18 or IFNγ, enhanced antibody production. The addition of IFNβ to IIV recapitulated key features of the live-vaccine cytokine program. IL-12 and IL-21 defined a parallel pathway independent of type I IFNs, with IL-12 inducing IL-21 in humans, unlike in mice. Delivery of IL-21 or IFNβ via mRNA lipid nanoparticles in vivo promoted long-lived plasma cell formation. Together, these findings define parallel pathways that regulate vaccine immunity. Our approach unites high-throughput organoid testing and human cohort studies, establishing a human-centric platform to identify adjuvant candidates.

## Abstract (web summary)

Chen and colleagues identify that baseline IFNβ levels correlate with antibody responses to inactivated influenza vaccines in humans, and show that type I IFNs, IL-12 and IL-21 functionally enhance antibody responses in human organoids.

## Main

Each year, approximately 800 million people receive the influenza vaccine, with 98% receiving the inactivated influenza vaccine (IIV). The elicited specific antibody response (protection against infection) varies widely, and the overall protection rate ranges from 20% and 60%, depending on the prevalent strains, with most morbidity and mortality occurring in older individuals (>65 years)^1^. Understanding the immunological factors that distinguish strong from weak responders can inform us about the potential mechanisms driving vaccine responses and suggest ways to improve them.

In our previous investigation of human cohorts, we found that while the specificity of the response after influenza vaccination was determined by host genetics^2^, the variability in the magnitude of this response was mostly driven by non-heritable factors^3^. Such factors may include previous cytomegalovirus infection and gut microbiota (for example, activating TLR5; refs. ^4,5^). Immune states at baseline or early vaccine responses may correlate and potentially regulate the vaccine response^6,7^. Multiple comprehensive studies conducted over the past decade have characterized the pre-vaccination or early post-vaccination immune transcriptome signatures that correlated with improved antibody responses to the influenza vaccine^7–10^. Several of these transcriptome signatures contained interferon (IFN)-inducible genes^7,8^; however, it has been unclear whether type I or type II IFNs were responsible for these signatures and if they were correlative or causal.

Here, we report the identification of multiple cytokines that correlate with elevated antibody responses in human influenza virus vaccination studies. We combined data from four influenza vaccine cohorts in five flu seasons (*n* = 581). We found a robust correlation between pre-vaccination (pre-VAX) serum cytokine abundance and post-VAX flu antibody responses in a subpopulation with lower baseline antibody titers (*n* = 389). Recently, we and others have developed *ex vivo* human immune cultures^11–13^. We have shown that human tonsil organoids can model the underlying mechanisms of vaccine responses, including somatic hypermutation and affinity maturation^12^. To distinguish between cytokines that correlate with an enhanced vaccine response and drivers of the response, we examined them in human tonsil organoids and spleen organoids. The immune organoids are primary tissue-derived cultures, distinct from many other organoids derived from tissue stem cells or induced pluripotent stem (iPS) cells. The immune organoids retain memory T and B cells from the donors, including those carrying memory against the influenza infections/vaccines. Meanwhile, the immune organoids recapitulate key features of the antibody response, including antigen-specific antibody production, somatic hypermutation, and affinity maturation^12,13^. We modified it to enable high-throughput screening and identified several cytokines that can enhance the antibody response elicited by the IIV in humans.

## Results

### Baseline antibody titers shape vaccine response

**Fig. 1:**
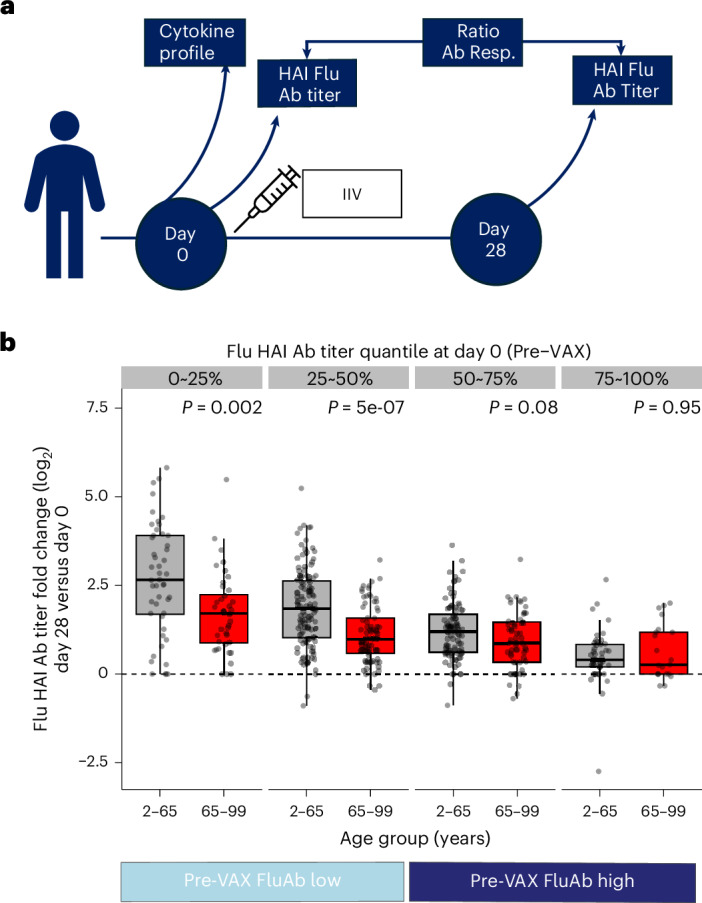
Age effects on vaccine responses were only detectable in individuals with low pre-vaccination flu antibodies. **a**, The scheme of the human cohort studies investigating the correlation between serum cytokine abundance and antibody response (ratio of HAI flu antibody abundance between day 28 and day 0), with individuals stratified by age group. **b**, The correlation between pre-VAX flu hemagglutinin inhibition (HAI) antibody titers and post-VAX antibody response. The antibody response was defined by the log_2_ ratio of the day 28 and day 0 flu HAI antibody titers. All participants who received a lower dose (15 µg per vaccine strain) IIV were included (while the older adults (>65 years) received the high-dose (60 µg per vaccine strain) formulation were excluded from the analysis due to dose confounding). Individuals were stratified into quartiles based on pre-vaccination (day 0) HAI titers. Pre-VAX low and high referred to the lower and upper 50% of pre-vaccination HAI titers, respectively; most in the low group had titers <40, a non-protective threshold. We performed Wilcoxon ranking tests between different age groups. All statistical tests were two-sided. **P < 0.01; ***P < 0.001. Source data

Given the central role of cytokines in modulating immune responses, we analyzed specific pre-vaccination (pre-VAX) serum cytokine profiles and the magnitude of the post-VAX antibody response (Fig. 1a). To achieve sufficient statistical power, we pooled data from four influenza vaccine cohorts across five seasons (Table 1). In these studies, IIV was administered to participants, and serum was analyzed at pre-VAX (day 0) and post-VAX (day 28) time points. Influenza (flu) hemagglutinin inhibition (HAI) antibodies against the vaccinated strains were measured at both time points, with the ratio defined as the antibody response. Considerable heterogeneity was present among cohorts in age, sex, flu strains, vaccine dose and cytokine profiling technology across seasons.

**Table 1:** Summary of influenza vaccine cohorts and sample characteristics

Cohort	Clinical Trials no.	Flu seasons					Age	Sex
		2009	2010	2013	2014	2015	Med (min–max)	(female %)
Flu vaccine study 15	NCT01827462	79	70	56	48	67	70 (22–90+)	56%
Flu vaccine study 17	NCT03020537	45	43	7	0	0	70 (8–90+)	64%
Flu vaccine study 18	NCT03022396	39	82	20	0	0	44 (2–89)	54%
Flu vaccine study 28	NCT03088904	0	0	0	25	0	22 (12–50)	72%
Total participants		*n* = 581					65 (2–90+)	58%

The correlation between pre-VAX (day 0) flu antibody titer and post-VAX (day 28) antibody response also showed great heterogeneity (Fig. 1b). To address this, we divided the population into four quartiles based on pre-VAX flu antibody titers. In line with previous studies, we observed that both the median and dynamic ranges of antibody responses decreased with an increasing abundance of pre-VAX antibodies, potentially due to the antigen clearance and thus lower vaccine ‘take’^7,14,15^. Aging is known to lower vaccine response. Of note, we found that responses were only lower in older individuals for participants with low pre-VAX flu antibodies (quantile 1 and 2, lower 50th percentile; Fig. 1b). These results suggested that the correlation between vaccine response and a biological characteristic, such as age, was considerably stronger in people with a low level of pre-VAX flu antibodies (and potentially higher vaccine ‘take’). Compared with the group with a high level of pre-VAX flu antibodies, the low-level group had a higher risk of flu infection^16,17^. Among the 389 participants with a pre-VAX flu HAI antibody titer lower than 40 (deemed not protected), 89% of them were in the group with a low level of pre-VAX flu antibodies. For this reason, our analysis focused on this group. These results identify low baseline antibody titer as a key context in which biological determinants of vaccine responsiveness are most readily detected.

### Baseline cytokines correlate with vaccine response

**Fig. 2:**
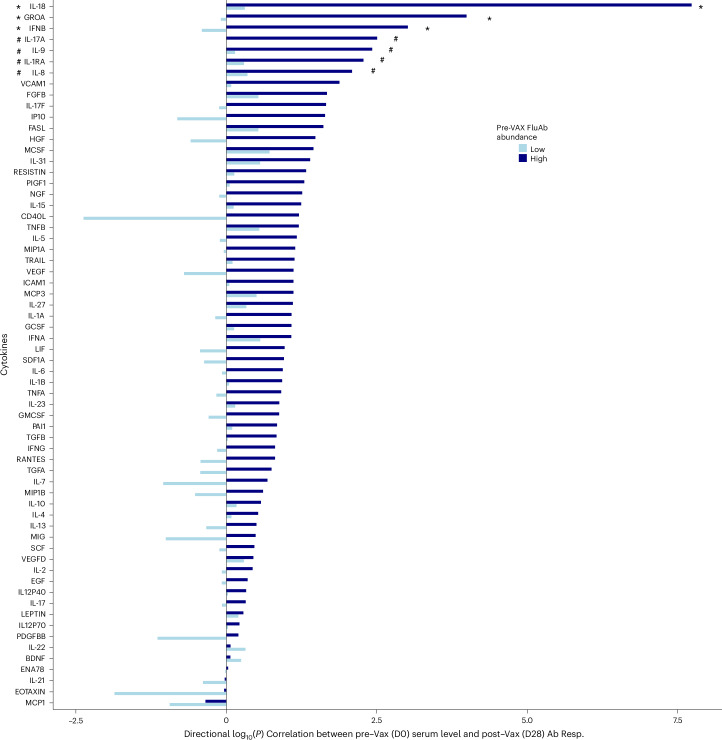
Meta-analysis identified a correlation between pre-VAX serum abundance of cytokines and post-VAX antibody response against the flu vaccine (IIV). We examined the correlation for 66 cytokines across the cohorts included in Table 1. The correlations were performed separately in groups with low or high pre-VAX flu HAI antibody (Ab) titers, as defined in Fig. 1b. log_10_ *P* values of the correlations were provided with the sign denoting the direction of the correlations (positive or negative slopes). FDRs were reported for significant or borderline significant hits. Random effect meta-analysis was performed to calculate effect sizes and standard errors; *z* tests were performed, all statistical tests were two-sided, and multiple-comparison adjustment was applied using the Benjamini–Hochberg FDR method. Exact FDR-adjusted *P* values can be found in Source Data Fig. 2. ^#^*P* < 0.05, *P < 0.1. Further methodological details are provided in the Supplementary Methods. Source data

We performed a meta-analysis of 66 cytokines measured across the four cohorts. The analysis identified two cytokines (IL-18 and IFNβ) and one chemokine (GRO-α, also known as CXCL1) whose pre-VAX serum abundance levels significantly correlated with post-VAX antibody response (false discovery rate (FDR) < 5%; Fig. 2). IFNβ and IL-18 activate distinct immune signaling pathways. IFNβ is a type I IFN that is critical for antiviral immunity, including antibody response. Among the three members of the type I IFNs tested (IFNα, IFNβ and IFNω), IFNβ has the highest binding affinity to the type I IFN receptor (IFNAR1/2), which is expressed by many cell types, including B cells. We also identified several cytokines with a borderline significant correlation (FDR < 10%), which included helper T (T_H_) cytokines IL-17 and IL-9 (Fig. 2).

### Human spleen organoids identify cytokine adjuvants

**Fig. 3:**
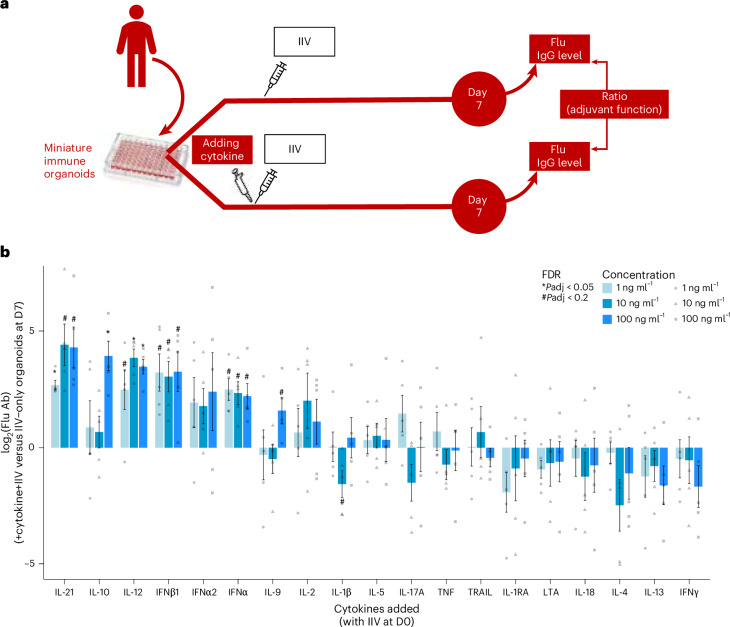
Functional screening in human immune organoids identified IIV-adjuvating cytokines. **a**, We tested the cytokine adjuvant function in three tonsil and two spleen organoids. We added 19 different cytokines together with the vaccine (IIV), and for each cytokine, we tested three different concentrations ranging from 1 ng ml^−1^ to 100 ng ml^−1^, except IL-1β (10× lower for each concentration) and IL-18 (10× higher for each concentration). We measured the antibody production (relative to non-cytokine-added IIV-only controls) on day 7. As the results from the tonsil and spleen organoids are similar (Extended Data Fig. 1), they are combined. **b**, The assay results are presented. Statistical significance was determined using a two-sided paired *t*-test across donors (*n* = 5) with FDR adjustment. Exact FDR values are provided in the associated data table. Source data

**Extended Data Fig. 1:**
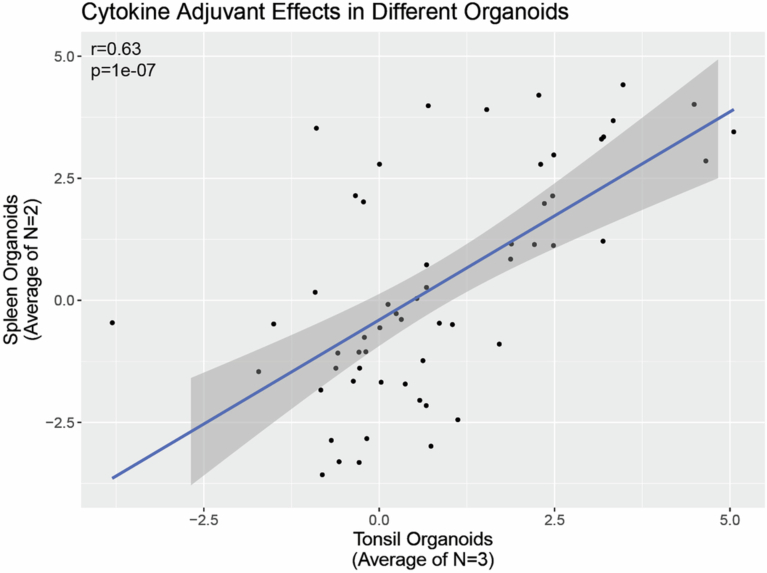
Cytokines demonstrated similar IIV-adjuvating effects in spleen and tonsil organoids. This figure is related to Fig. 3. We harvested 3 tonsils and 2 spleens from donors and vaccinated the organoid cultures with IIV. We added 19 different cytokines together with the vaccine (IIV), and for each cytokine, we tested 3 different concentrations ranging from 1 ng/ml to 100 ng/ml. We measured the antibody production (relative to non-cytokine-added IIV only controls) on day 7. The adjuvant effects of 19 cytokines at 3 different concentrations (a total of 57 points) are plotted on the scatter plot, with the x-axis representing the averaged value for tonsil organoids and the y-axis representing the spleen organoids. The Pearson’s correlation coefficient and p values are listed. Source data

Human cohort studies can identify correlations of immune function, but they cannot prove causality without interventional studies. An individual interventional study in a mouse model typically tests only one or two cytokines. When cytokine adjuvant effects were compared across mouse studies, there was heterogeneity in cytokine delivery formats (DNA or protein), antigen and delivery routes (such as intramuscular, intranasal and intraperitoneal; Supplementary Table 1). A functional systems immunology approach that compares different molecules delivered in the same format and assayed against the same antigen in a human culture system can allow quantitative evaluation of adjuvant effects and prioritize the adjuvant candidates. Building on our previous work, we devised a low-cell-input immune organoid culture system (reducing the cell input by 97% to 1.6 × 10^5^ cells), which enabled us to generate many immune organoid cultures in a 96-well format. We performed a screen and systematically examined cytokine adjuvant functionality to the inactivated vaccine using a functional systems immunology approach (Fig. 3a). We collected three tonsils from patients from the clinic. In collaboration with Donor Network West, an organ-processing organization that serves Northern California and Northern Nevada, we procured spleens from two authorized ventilated deceased donors. Using this higher throughput immune organoid format, we generated five sets of immune organoids from five different participants. We administered them with IIV, alone or in combination with 19 different cytokines. Most of these cytokines have been shown to boost antibody response in one or multiple mouse vaccination models (Supplementary Table 1). We combined the data as the cytokines’ adjuvant effects are similar between spleen and tonsil organoids (Extended Data Fig. 1). For each cytokine, we tested three different concentrations, ranging from 1 ng ml^−1^ to 100 ng ml^−1^; however, for IL-1β (ten times lower at each concentration) and IL-18 (ten times higher at each concentration), we used concentrations different from those of other cytokines, as the physiological concentrations in human serum differ from those of other cytokines^18^. Antibody production (relative to the IIV-only controls) on day 7 (Fig. 3a) was measured. In total, we assayed 58 different conditions, including the control, across five biological replicates. The screen revealed that all type I IFNs (IFNβ and IFNω, though IFNα was less effective) enhanced antibody production induced by IIV (Fig. 3b). Moreover, we discovered a potent adjuvant function of several other cytokines. Among them, IL-21 and IL-9, which are secreted by T follicular helper (T_FH_) and T_H_9 cells. IL-12 induces the formation of T_H_1 and T_FH_ cells. IL-10 can act as one of the downstream effectors of type I IFNs. For most factors, the effect was detected at concentrations as low as 1 ng ml^−1^. Notably, IL-18 or its downstream effector, type II IFN (IFNγ), did not enhance the IIV-induced antibody response in human spleen and tonsil organoids (Fig. 3b), which contrasts with the strong correlation observed between IL-18 abundance and antibody response in human cohorts. These findings show that human immune organoids can functionally distinguish cytokines that directly enhance inactivated vaccine-induced antibody responses from those correlative factors.

### Type I IFNs mimic live-vaccine responses

**Fig. 4:**
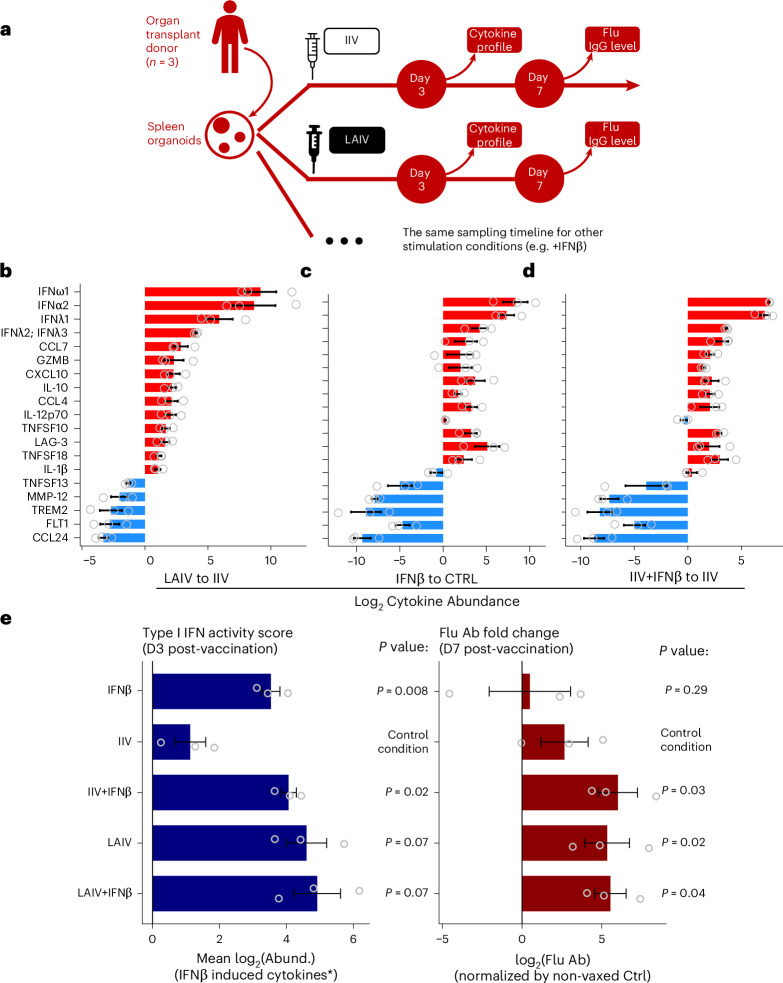
Type I IFNs drive an immune activation program distinguishing the response induced by live versus inactivated vaccines. **a**, We stimulated the spleen organoids derived from three transplant organ donors with IIV, LAIV, IFNβ or combinations (see below). **b**–**d**, Cytokines differentially induced between stimulation conditions. Cytokines elevated between stimulation conditions (LAIV versus IIV; IFNβ versus control (not vaccinated); IFNβ versus control (IIV-vaccinated)) are shown. Cytokines met significance criteria in the LAIV versus IIV comparison are shown (*P* < 0.05 and fold change ≥ 2). Differential cytokine abundance was assessed using a two-sided paired limma analysis (moderated *t*-test using empirical Bayes shrinkage) across donors (*n* = 3). Error bars represent standard error. **e**, Type I IFN activity and flu antibody responses of organoids under different stimulation conditions are shown. Statistical significance was determined using two-sided paired *t*-tests across donors (*n* = 3) and the corresponding *P* values are reported. Source data

**Extended Data Fig. 2:**
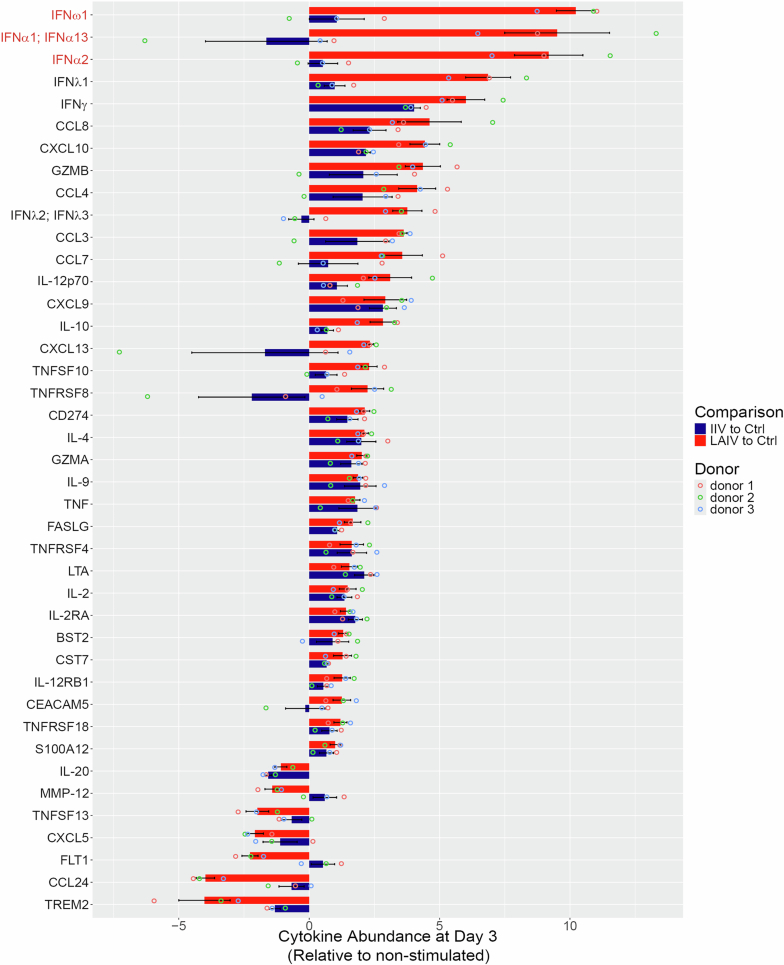
The cytokine profiles induced by IIV or LAIV in spleen organoids. This figure is related to Fig. 4. We measure the cytokine profiles using supernatants of the spleen organoids (N = 3) culture at day 3 post-VAX. All cytokines significantly altered after vaccination by either vaccine (as compared with the non-stimulated) are shown. Red texts highlight the type I interferons (IFNs).

**Extended Data Fig. 3:**
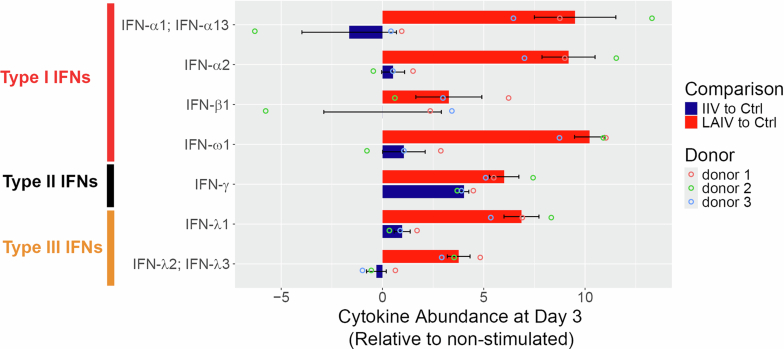
IFNs induced by IIV or LAIV in spleen organoids. The figure is related to Fig. 4. The abundances of type I/ II/ III cytokines in the supernatants of spleen organoids stimulated by either IIV or LAIV were reported.

While cytokines can regulate vaccine response, vaccination can also induce cytokines that coordinate the antibody response. We exposed spleen organoids to IIV, and collected the supernatant on day 3, analyzing the samples using a DNA-barcoded multiplexed cytokine detection technology with femtomolar sensitivity (NULISA) (Fig. 4a). Of the 250 cytokines/chemokines assayed, type II IFN (IFNγ) was the most significantly induced (Extended Data Fig. 2). Notably, none of the type I IFNs (IFNα/β/ω) were induced by IIV (Extended Data Fig. 3).

**Extended Data Fig. 4:**
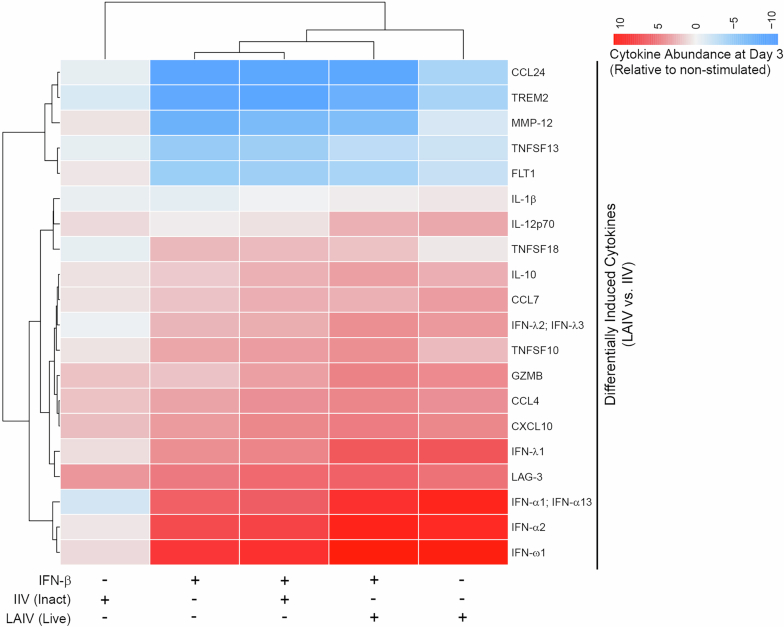
The cytokine profiles of spleen organoids under different stimulation conditions. This figure is related to Fig. 4. We measured the cytokine profiles using supernatants of the spleen organoids (N = 3) culture at day 3 post-VAX. Cytokines differentially induced by LAIV versus IIV (FDR < 0.05 and fold change >2) are shown.

Next, we examined the cytokine-induction profile of another type of influenza vaccine, the live attenuated influenza vaccine (LAIV). Unlike the inactivated format, the live vaccine contains a mutated influenza virus capable of completing a few cell cycles. Both IIV and LAIV are US Food and Drug Administration (FDA)-approved (different age groups and administration routes). The cytokine-induction profiles of the LAIV and IIV share a strong induction of T_H_2 cytokines (IL-5, IL-13 and IL-4), T_H_9 cytokine (IL-9) and type II IFN (Extended Data Figs. 2 and 3); however, the LAIV also induced a unique and prominent type I and III IFN response (Extended Data Fig. 3), with concentrations up to ~1,000 times (10 log_2_) higher than IIV (Fig. 4b), along with a broad spectrum of other cytokines, including IL-10, IL-12, CCL-7 and CXCL-10. (Extended Data Fig. 4).

To examine the regulation of other cytokines by type I IFN, we added IFNβ to the non-vaccinated spleen organoid controls. Notably, the cytokine profile induced by IFNβ almost overlapped with the cytokines differentially induced between the live and inactivated vaccines: 13 of 14 LAIV-IIV differentially upregulated cytokines were also induced by IFNβ. In comparison, four of five LAIV-IIV differentially downregulated cytokines were also suppressed by IFNβ (Fig. 4c). Similar cytokine-induction profile overlaps occurred when we added IFNβ into the IIV-vaccinated controls and compared them (Fig. 4d). As a result, the cytokine profile of the organoids treated with IFNβ phenocopied the live-vaccine-treated samples organoids (Extended Data Fig. 4).

When we added IFNβ to the spleen organoids, it induced the expression of other type I IFNs (IFNα and IFNω; Fig. 4c,d), which bind to the same receptor (IFNAR1/2) and induce the same signaling pathway, suggesting a positive feedback loop between the induction of type I IFNs. Additionally, type I IFNs seem to be a key regulator of the live-vaccine-specific cytokine profile, with their abundance differentiating the inactivated versus live-vaccine responses (also including the antibody response, see below). Next, we examined the functionality of type I IFN (IFNβ) to adjuvant antibody responses. Due to the existence of multiple type I IFNs, the concentration of a single protein could not represent the type I IFN activity. We measured the global type I IFN activity by taking the geometric mean of the abundance of all cytokines significantly induced by IFNβ. The type I IFN activity scores showed that the IIV was defective in inducing type I IFN activity, which was rescued by IFNβ addition (Fig. 4e). While the LAIV efficiently induced type I IFN activity, adding more IFNβ did not further enhance it, suggesting saturation (Fig. 4e). Of note, the antibody production on day 7 post-VAX rose alongside the type I IFN activity upon antigen challenge (Fig. 4e). Adding type I IFN (IFNβ) reshaped the downstream global cytokine profile to the live-vaccine-like state. Together, these data identified type I IFN as a central regulator that shifts the inactivated-vaccine response toward a live-vaccine-like state.

### IFNβ regulates low-dose responses in older adults

**Extended Data Fig. 5:**
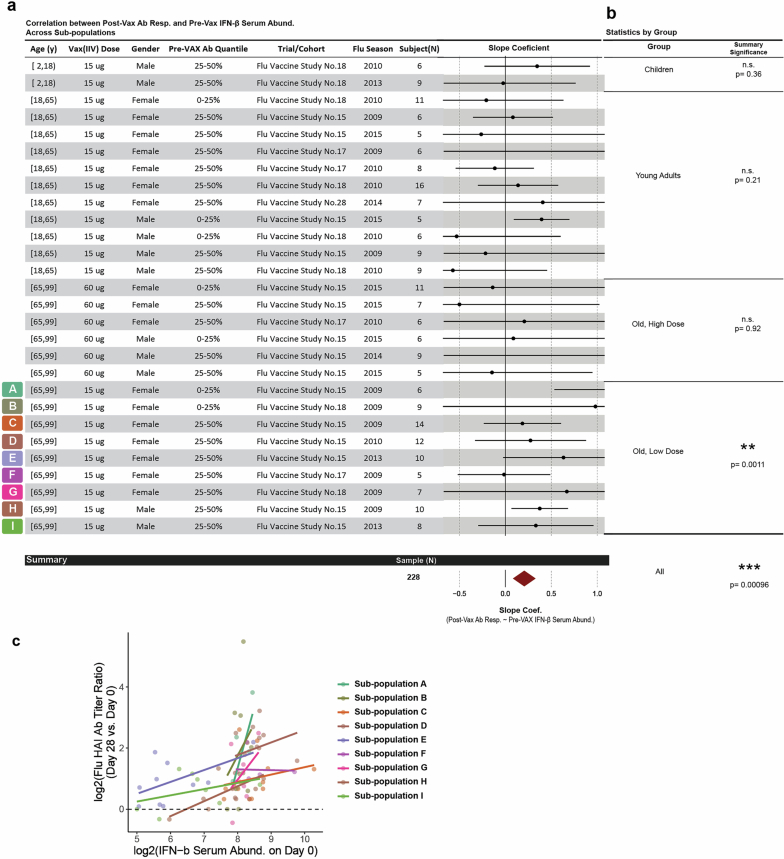
Pre-VAX IFNβ serum abundances correlate with Post-VAX antibody responses in the older adults (>65 y) receiving low-dose flu vaccines. We divided the dataset by confounders (age, vaccine dose, gender, day 0 flu antibody quantile, cohort, flu season) into 28 subpopulations and performed correlations with each subpopulation. **a**) The left panel described the characteristics of each one of the 28 subpopulations. The slope coefficients panel described the correlation within each subpopulation, with summary statistics provided in the bottom. **b**) We summarized the 28 subpopulations into 4 groups by age and vaccine doses. For each group, summary statistics are provided. Details about the correlation and meta-analysis can be found in the Supplementary Methods. **c**) The correlation between IFNβ serum abundance and antibody response in the old receiving a low-dose IIV. The subpopulations are marked accordingly in a. The IFNβ serum abundance data are not normalized here, with the batch effects visible. Also see Extended Data Fig. 6. Source data

**Extended Data Fig. 6:**
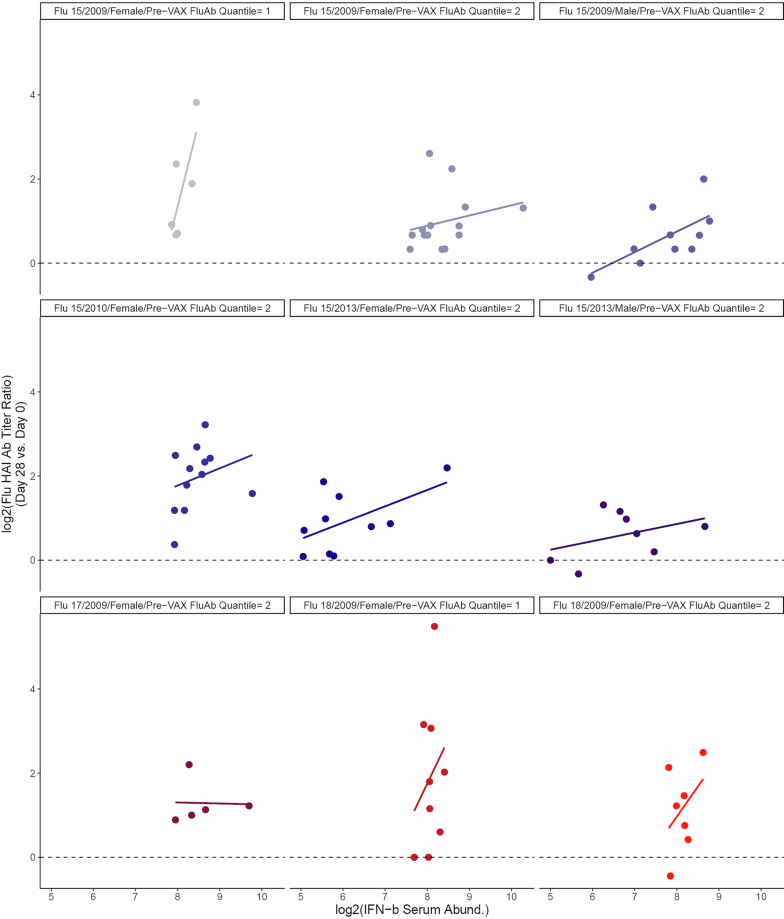
The correlation between pre-VAX IFNβ serum abundance and post-VAX flu antibody responses in the older adults (>65 y) receiving a low-dose flu vaccine. This figure is related to Fig. Extended Data Fig. 5c. The correlations (trends highlighted by the lines) of the 9 subpopulations in the ‘Old, low dose’ group (Fig. Extended Data Fig. 5b, lower) are plotted. The title of each panel corresponds to a subpopulation listed in Extended Data Fig.5a.

The association studies in human cohorts and functional studies in tonsil and spleen organoids support the view that IFNβ is a natural adjuvant underlying variability in flu vaccine responses in humans. Interestingly, we found considerable heterogeneity correlation with post-VAX antibody response and IFNβ across the subpopulations (Extended Data Fig. 5a). The heterogeneity was primarily due to age differences, with the correlation only reaching significance in older adults (>65 years). Among older adults, the correlation was more pronounced in those who received low-dose vaccines. When the low dose was found suboptimal for older adults^19^, the cohort studies followed the most current knowledge and switched the older adults to a version with high-dose antigen in the 2014–2015 season. Concurrent to this change, the association between baseline IFNβ concentration and antibody response disappeared (Extended Data Figs. 5b,c and 6).

**Extended Data Fig. 7:**
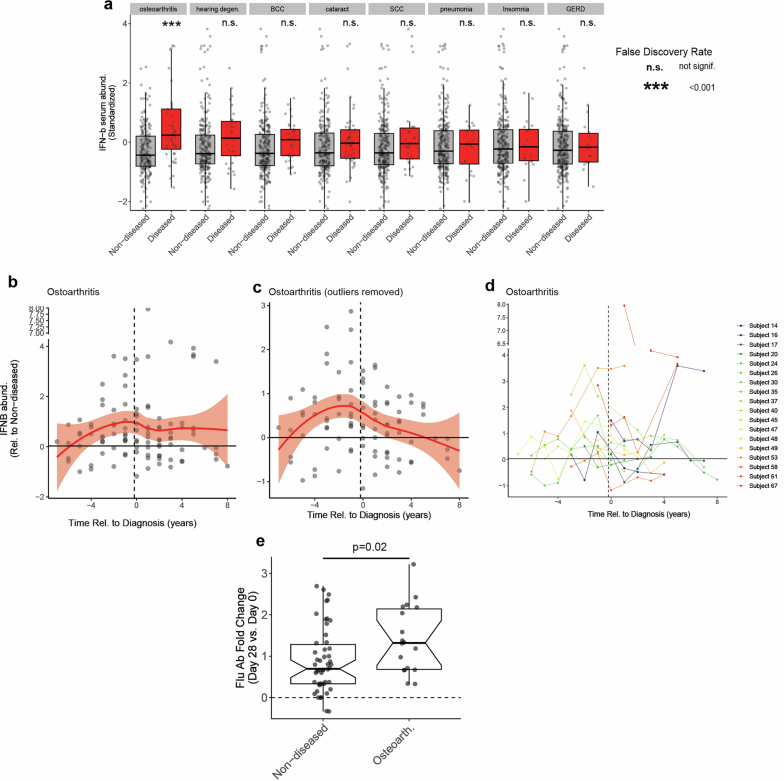
Osteoarthritis incidence modifies the IFNβ abundance and vaccine response. The figure is related to Extended Data Fig. 5. We examined the correlation between disease incidence, IFN-β serum abundance, and flu vaccine antibody response in a longitudinal cohort (Flu Study 15, one of the 5 cohorts in Fig. 1a). This cohort collected medical histories annually. The analysis was conducted among older adults (>65 years). **a**) The IFNβ serum abundance between the diseased and non-diseased samples. The diseased samples are samples collected within (+/−) 2 years relative to a diagnosis. The non-diseased samples are from subjects without a medical history of the disease. Wilcoxon ranking tests were performed for the comparisons. **b**–**d**) Temporal profile of IFN-β serum abundance relative to the diagnosis of osteoarthritis within the cohorts. The loess smooth line is provided with confidence intervals. **c**) The outliers (standardized abundance >=3) were removed, and the temporal correlation was retained. **d**) the temporal profile of individual patients diagnosed with osteoarthritis during the study. **e**) The flu HAI antibody responses (day 28 vs. day 0 ratio) in non-diseased and osteoarthritis subjects. Similar to Figs. 2 and 3, the analysis was performed in subjects with a low level of pre-VAX flu antibodies (Fig. 1c). Refer to the Supplementary Methods. Source data

The age-dependent correlation between pre-VAX IFNβ abundance and post-VAX response prompted the hypothesis that certain aging-associated conditions may underlie variation in IFNβ abundance and vaccine response in this population. The Stanford–Ellison Cohort (flu vaccine study 15; Table 1) surveyed medical history annually and recorded 648 clinical events from 135 participants, 628 of which occurred in older adults (>65 years). Among them, nine types of clinical events accumulated more than ten incidents in the cohort. We asked whether the serum IFNβ abundance changed within 2 years (before or after) a disease diagnosis. The strongest association identified is from osteoarthritis (Extended Data Fig. 7a). Osteoarthritis is a highly prevalent condition in older adults, with 20 incidences in the cohort (compared to three cases of rheumatoid arthritis). We leveraged the longitudinal nature of the study and examined the temporal correlation between serum IFNβ abundance and osteoarthritis incidence. The IFNβ abundance was significantly elevated from the population mean up to 4 years before diagnosis and lasted up to 5 years after (Extended Data Fig. 7b–d). The temporal correlation was robust and retained significance after outliers were removed (≥3 × s.d. from the population mean) (Extended Data Fig. 7c). Within the time frame, we found that the antibody response to the influenza vaccine was significantly elevated in participants with osteoarthritis (Extended Data Fig. 7e). Thus, a highly prevalent aging-associated condition, osteoarthritis (including its preclinical stage), was a modifier of the IFNβ abundance and vaccine response. These findings identify IFNβ as a selective regulator of vaccine responsiveness in older adults receiving lower antigen doses.

### IL-12 and IL-21 define the live-vaccine pathway

**Fig. 5:**
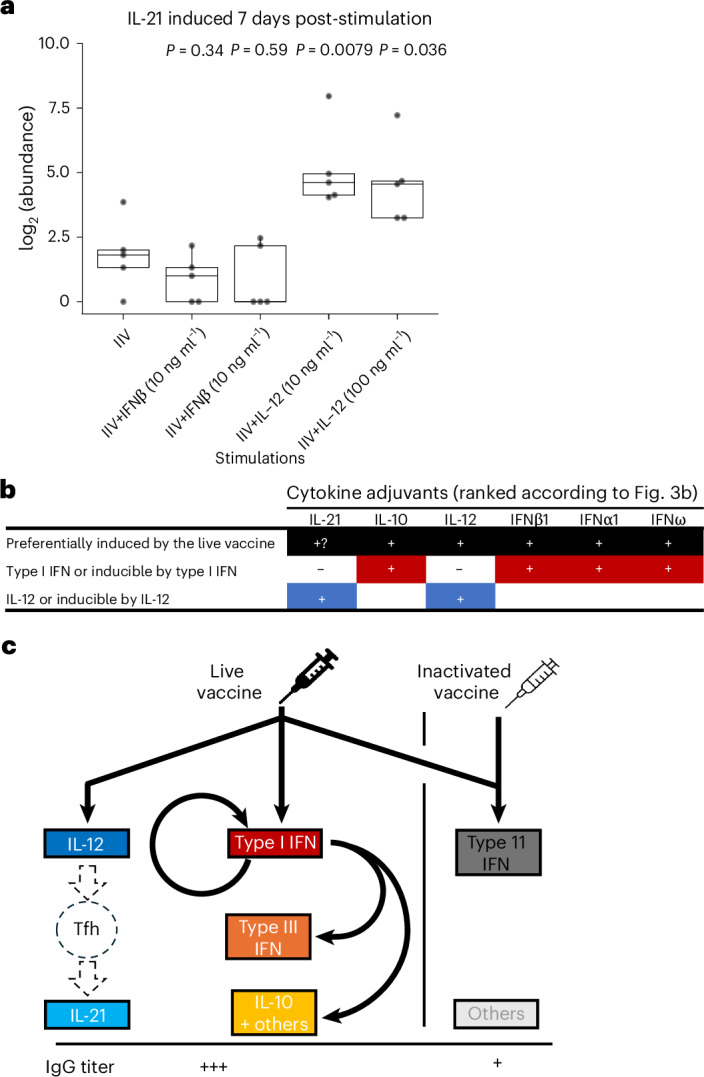
Two distinct cytokine pathways regulating vaccine responses in humans. **a**, IL-21 abundance measurement 7 days post-stimulation. **b**, The induction of cytokine adjuvants (capable of boosting vaccine responses, Fig. 3b). The induction profile is based on data in **a** and Fig. 4c,d. While the NULISA assays for the live vaccine in Fig. 4 did not cover the IL-21 measurement, it is known that the live vaccine preferentially activates its main producer (T_FH_) in human immune organoids. **c**, The model describes the different immune activation programs induced by the live and the inactivated influenza vaccines. We performed Wilcoxon ranking tests between different age groups. Wilcoxon rank-sum test (two-sided) was used. Sample size *n* = 5 (three tonsil donors and two spleen donors). Box plots show median (center line), interquartile range (box) and whiskers representing the full data range.*P < 0.05; **P < 0.01.

In the immune organoid screen, we identified numerous cytokine adjuvants capable of boosting antibody response. Of note, most cytokine adjuvants were induced to higher levels by the LAIV than by the IIV (Fig. 4b). This highlights the important role of cytokines induced by the live vaccine. The four cytokine adjuvants are either type I IFN (IFNα, IFNβ or IFNω) or induced by type I IFN (IL-10) (Fig. 4c,d); however, two potent cytokine adjuvants, IL-12 (Fig. 4c,d) and IL-21 (Fig. 5a,b), are not induced by type I IFN in human immune organoids, and were identified as live-vaccine-specific cytokines outside of the type I IFN pathway. Previous studies have shown that IL-12 is a potent human cytokine at inducing IL-21-expressing CD4^+^ T cells, which are responsible for B cell ‘help’^20^. (Fig. 5a). These data support the existence of at least two cytokine pathways regulating human vaccine responses (Fig. 5c). The IL-12–IL-21 pathway operates in parallel with type I IFN.

### mRNA LNPs enhance vaccine responses in mice

We quantitatively compared 19 cytokines and found that IFNβ and IL-21 demonstrated the most potent adjuvant function at the lower concentration (1 ng ml^−1^). We next tested the functional relevance of these cytokine pathways in vivo using a murine model. To this end, we employed an antigen-coding messenger RNA lipid nanoparticle (mRNA LNP) platform in mice immunized with IIV. The mRNA component of mRNA LNPs is known to induce a strong type I IFN response, while its LNP component promotes the activation of T_FH_ cells^21^, the primary source of IL-21.

**Fig. 6:**
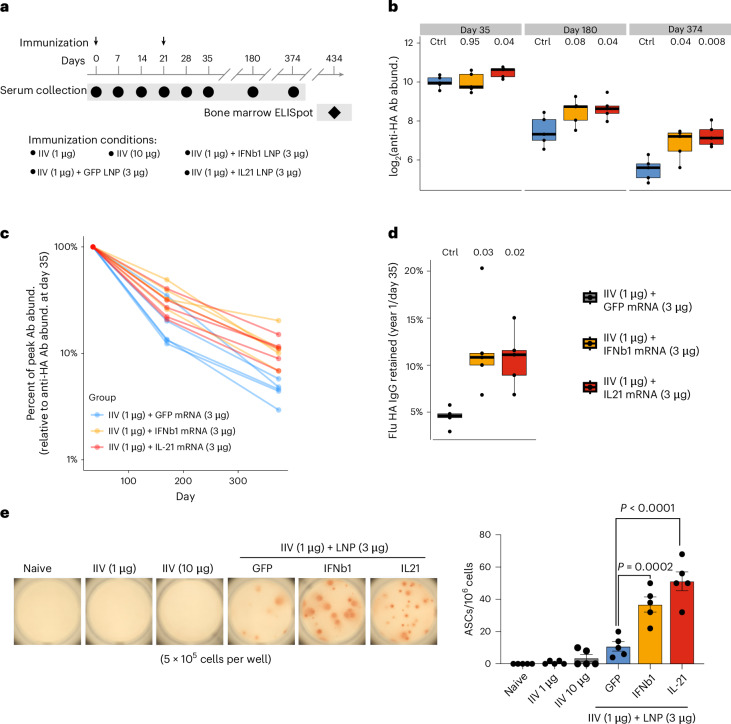
mRNA LNP cytokine adjuvants enhance the durability of antibody responses to inactivated influenza vaccination. **a**, The experimental scheme illustrates testing of the adjuvant functionality of mRNA lipid nanoparticles (mRNA LNPs) encoding GFP or cytokines. Mice were primed with IIV on day 0 and boosted on day 21 as indicated, with serum collected at the indicated time points through day 374 for antibody analysis and bone-marrow cells collected on day 434 for ELISpot analysis; immunization conditions are listed. **b**, Serum anti-HA IgG binding intensities against influenza HAs from in-vaccine strains were quantified by a custom Luminex bead assay, and the geometric mean is taken. Vaccination groups are color-coded as shown in **d**. **c**, Longitudinal trajectories of anti-HA IgG abundance over extended follow-up are shown for individual mice and expressed as the percentage of peak antibody levels relative to day 35. **d**, Anti-HA IgG retention over long-term follow-up is shown as the percentage of antibody levels on day 374 relative to day 35. **e**, ASC responses in the bone marrow are shown by representative ELISpot wells and quantification of influenza-specific IgG ASCs per 10^6^ cells measured on day 434 following immunization with IIV alone or IIV combined with mRNA LNPs encoding GFP, IFNβ, or IL-21; naïve mice are shown as a negative control and each dot represents an individual mouse. All panels show data from *n* = 5 mice per immunization group (biological replicates). Data are shown as mean ± s.e.m.; box plots (**b**,**d**) indicate median (center line), interquartile range (box), and whiskers representing the full data range. For panels **b**–**d**, two-sided *t*-tests were performed with FDR-adjusted *P* values shown. For **e**, statistical significance was determined using one-way analysis of variance with multiple comparisons adjusted. Source data

To evaluate this approach, we administered mRNA LNPs encoding green fluorescent protein (GFP) in combination with IIV in mice (Fig. 6a). As GFP is a foreign protein to mice, it is relatively immunologically inert when delivered alongside micrograms of influenza antigen, allowing us to confirm successful antigen expression via fluorescence imaging. Mice were boosted on day 21 after the primary immunization, and serum samples were collected at multiple time points for antibody profiling. The IIV formulation included hemagglutinins (HAs) from human influenza strains (H1N1, H3N2 and B) circulating during the 2023–2024 season. Serum antibody responses were analyzed using a custom Luminex panel that detects antibodies against both in-vaccine (on-target) and out-of-vaccine (crossreactive) HAs, including zoonotic strains such as H5N1 and H17N10.

**Extended Data Fig. 8:**
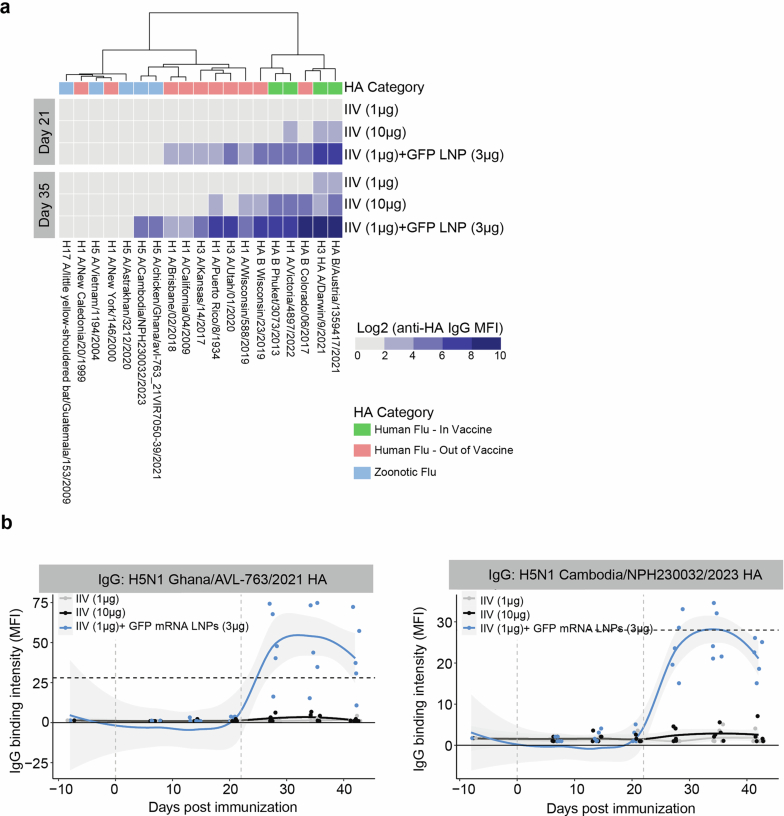
Kinetics and antigenic breadth of IgG responses to H5N1 HA following IIV immunization with or without mRNA LNP adjuvant. **a**) Heatmap showing serum IgG levels (quantified as IgG binding intensities, Log2 MFI) against a diverse panel of influenza hemagglutinins (HAs), measured by custom Luminex bead assay at day 21 and day 35. HA antigens are categorized as Human Flu–In Vaccine, Human Flu–Out of Vaccine, or Zoonotic Flu, as indicated by the top color bar. **b**) Serum IgG binding intensities (MFI) against recombinant H5N1 HA proteins from A/chicken/Ghana/AVL-763/2021 (top) and A/Cambodia/NPH230032/2023 (bottom) were quantified over time using a custom Luminex assay. Mice were immunized as shown in Fig. 6a. The two vertical dashed lines indicate the timing of the prime and boost immunizations. The horizontal dashed line represents the IgG response (MFI) against in-vaccine influenza strains induced by an unadjuvanted high-dose vaccine (10ug) at day 35, whose antigen dose is 10 times that of the adjuvanted condition.

At day 21 post-primary immunization, 1 μg IIV alone failed to elicit detectable antibody responses, whereas 10 μg IIV induced moderate titers against the vaccine, including in-vaccine HAs (B/Austria/1359417/2021, H3 A/Darwin/9/2021, and H1 A/Victoria/4897/2022; Extended Data Fig. 8a). In contrast, 1 μg IIV combined with GFP mRNA LNP markedly enhanced antibody titers against all in-vaccine HAs and exceeded those elicited by 10 μg IIV alone. In addition, this group exhibited crossreactive antibody responses to out-of-vaccine HAs. By day 35 (14 days post-booster), antibody titers increased further. The 1 μg IIV-alone group showed low but detectable antibody responses against two in-vaccine HAs B/Austria/1359417/2021 and A/Darwin/9/2021 (H3N2), whereas the 10 μg IIV group produced substantially higher titers to all in-vaccine strains, along with modest crossreactivity. Notably, the group that received IIV plus GFP mRNA LNP showed the highest overall antibody titers, including responses to zoonotic HAs. The crossreactive antibody titer against the two most recent H5N1 strains in this group was comparable or higher than the antibody titer against in-vaccine strains induced by the high-dose non-adjuvanted influenza vaccines (10 μg) (Extended Data Fig. 8b). Even with an immune-irrelevant encoded protein, mRNA LNPs boosted the quantity and the breadth of humoral responses to inactivated vaccines.

**Extended Data Fig. 9:**
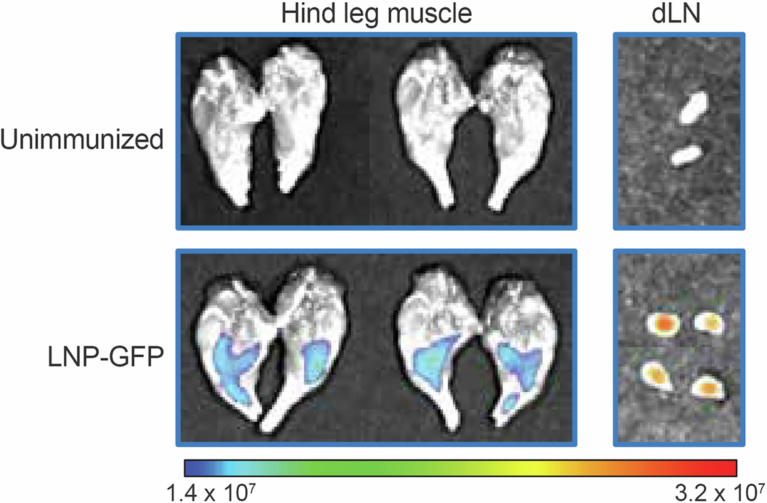
Fluorescence imaging of GFP in hind leg muscles and inguinal draining lymph nodes (dLNs) from unimmunized and LNP-GFP immunized mice. The images were taken 24 hours after injection. Data are representative of two independent experiments.

To examine the tissue localization of mRNA LNP activity, we tracked GFP tissue distribution 24 h post-injection. Fluorescence was detected at both the injection sites, muscles and draining lymph nodes, with substantially higher intensity in the lymph nodes (Extended Data Fig. 9). This suggested efficient mRNA delivery and protein expression in secondary lymphoid tissues. This supports that cytokines encoded by mRNA LNPs may act as an adjuvant in lymph nodes to modulate vaccine response. Thus, we tested whether mRNA LNPs encoding specific cytokines could enhance vaccine responses beyond the innate adjuvanticity of LNPs themselves (Fig. 6a). Mice were immunized with 1 μg IIV in combination with mRNA LNPs encoding either mouse IFNβ or IL-21. At day 35, inclusion of IL-21 mRNA LNPs outperformed GFP mRNA LNPs in boosting antibody titers against both in-vaccine HA antigens (Fig. 6b). At the same time, IFNβ mRNA LNPs showed no advantage at this timepoint. At 12 months post-immunization, both IL-21 and IFNβ mRNA LNP resulted in ~five times higher antibody titers than the GFP mRNA LNPs (Fig. 6c). Comparison of titers between 12 months and day 35 revealed that either IL-21 or IFNβ mRNA LNPs slowed the waning of antibody response (Fig. 6d). The enhanced antibody durability was accompanied by much higher rates of antigen-specific long-lived plasma cell formation induced by either cytokine mRNA LNPs, as shown by bone-marrow B cell ELISpot assays (Fig. 6e). The data suggest that cytokine mRNAs sustained antibody production by enhancing long-lived plasma cell persistence.

These results demonstrate that cytokines IFNβ and IL-21 can serve as effective vaccine adjuvants to enhance antibody responses. Using mRNA LNPs to deliver these cytokines in vivo provides a flexible strategy to boost the immunogenicity of inactivated vaccines.

## Discussion

Identifying baseline predictors of an individual’s vaccine response is a key goal for systems vaccinology. Here, we identify several cytokines (IL-18, GROα/CXCL1 and IFNβ) whose pre-vaccination abundance correlates with subsequent antibody responses to influenza vaccination. Notably, these relationships were most evident in individuals with low pre-VAX HAI flu antibody titers. The finding is consistent with previous observations that this population launched more robust interferon responses following vaccination, potentially due to reduced vaccine antigen clearance and thus greater vaccine ‘take’. Previous studies have linked transcriptomic signatures, particularly interferon-related programs, to vaccine outcomes^7,15,22^. Our findings suggest that cytokines may act as upstream drivers of these signatures. These results highlight the importance of baseline immune stratification for uncovering determinants of vaccine responsiveness and suggest that such stratification strategies may be broadly applicable for identifying predictive immune signals in vaccine studies.

Combining correlative and interventional studies, we found that IFNβ is a natural adjuvant capable of boosting antibody response in the human population. The IFN-related gene expression signature (induced by either type I or II IFN) correlated with day 28 antibody responses^7,8^. A previous study reported a shared transcriptome signature between vaccine high responders in patients with systemic lupus erythematosus, a disease characterized by chronic activation of type I IFN signaling^9^. Higher type II IFN production (by CD8^+^ T cells) correlates with influenza vaccine responses in COVID-19 recoverees^23^. Also, patients with systemic lupus erythematosus who did not respond to the SARS-CoV-2 vaccine had minimal IFNγ responses following a booster^24^. The relative contribution of type I or type II IFNs to vaccine responses has not been tested in a human immune model. Here, we found that type I IFNs, but neither type II IFN nor IL-18 (a potent inducer of type II IFN), could enhance influenza vaccine responses. Adding a single cytokine, type I IFN (IFNβ), recapitulates most of the live-vaccine-specific immune activation program, including other type I IFNs and another cytokine adjuvant, IL-10 (but not IL-12 or IL-21). Moreover, adding type I IFN boosted the inactivated vaccine’s antibody production to a level similar to that of the live vaccine. Moreover, we identified type I IFN as an immune regulator that selectively influences vaccine responsiveness in a high-risk group. Older adults bear a disproportionate burden of influenza-associated morbidity and mortality^17^ and consistently experience suboptimal vaccine-induced antibody responses^25^.

Our systemic screen identified a second pathway (IL-12/IL-21) that is type I IFN independent. Despite profound defects in the innate antiviral defense, individuals with type I IFN deficiency can still mount humoral responses. For example, YFV-neutralizing antibodies in *IFNAR1*-deficient patients can develop following vaccination and exposure^26^, indicating that B cell priming and antibody production can proceed independently of intact type I IFN signaling. In human spleen organoids, IL-12 and IL-21 were not induced by type I IFN stimulation, whereas IL-21 was induced by IL-12. Of note, IL-12 can regulate B cell function directly^27^. Together, these findings support a model in which IL-21/IL-12 operates independently of type I IFN signaling. The interaction of the orthogonal cytokine pathways in the breadth of antibody responses warrants further investigation.

To test whether the cytokine pathways identified here could be leveraged in vivo, we evaluated cytokine delivery using mRNA LNP. Numerous cytokines have been evaluated as adjuvants in protein form in preclinical models (Supplementary Table 1). mRNA LNP, a potent inducer of both type I IFN and IL-21, augmented the durability of the antibody response. Intriguingly, IFNβ mRNA LNP did so without further increasing the peak antibody titer (compared to GFP mRNA LNP at 14 days post-vaccination). While mRNA LNP has been widely used to encode antigens (such as in COVID-19 vaccines), its potential for delivering cytokine adjuvants has not been fully explored. A recent study reported that mRNA LNP encoding IL-12 enhanced the vaccine response to COVID-19 antigens^28^. Both IL-21 and IL-12 showed potent adjuvant effects; however, the lipid components of mRNA LNP vaccines, particularly the ionizable and PEGylated lipids, can induce potent and broad inflammatory effects. Future studies should develop immunologically inert lipids to improve precision and safety.

Our study highlights differences between human and mouse vaccine responses. Studies in mice show that IL-12 does not induce mouse CD4^+^ T cells to express IL-21 (refs. ^29,30^), whereas IL-12 strongly induces IL-21 expression in human spleen organoids, consistent with previous studies in human CD4^+^ T cells^31,32^. Numerous cytokines (Supplementary Table 1) have been reported to enhance antibody responses to influenza vaccination in murine models, but many of them did not have this effect in our organoid system. These discrepancies may reflect differences between recall responses in humans versus primary responses in mice, as well as broader species-specific effects, which are worthy of further investigation. While animal testing remains the cornerstone for drug development, it has limited predictive value for efficacy in humans^6,33^. Recent US FDA plans to adopt ‘human-relevant methods’ for drug evaluation emphasize this need. Our approach unites high-throughput organoid testing and human cohort studies, establishing a human-centric platform to identify adjuvant candidates.

## Methods

Written informed consent was obtained from all study participants and the study protocol was approved by the Stanford University Administrative Panels on Human Subjects in Medical Research (Institutional Review Board (IRB)-62436), confirming compliance with relevant ethical regulations.

### Human samples

Human samples used in this study were derived from multiple sources. The influenza vaccine cohort included *n* = 581 participants, with ages ranging from 2 to 90+ years and a sex distribution of 58% female, as summarized in Table 1 and Fig. 1b. Human immune organoid experiments were performed using primary tissues from independent donors, including *n* = 5 donors (three tonsil and two spleen donors) for cytokine screening experiments and *n* = 3 spleen donors for cytokine profiling experiments (Fig. 4). Donor demographic information, including age and sex, was not available for all organoid samples. Donor demographic information, including age, sex and ethnicity, was available for all organoid donors and is provided in Supplementary Table 2. Cause of death information is provided for deceased donors (spleen samples) and does not apply to tonsil donors.

### Cohort design and data retrieval

We retrieved human serum cytokine and antibody data from previous influenza vaccine studies conducted by the Stanford Human Immune Monitoring Center (HIMC). Only individuals who received non-adjuvanted, inactivated influenza vaccines (Fluzone, standard or high dose) were included; individuals who received FluMist (live attenuated) or Fluad (MF59-adjuvanted) vaccines were excluded. Various laboratories measured HAI antibody titers between 2007 and 2015. To ensure data quality across years, we analyzed a longitudinal cohort (flu vaccine study no. 15) to assess year-to-year consistency. The 2015 measurements (conducted by a CDC-accredited laboratory) were treated as the gold standard, and pre-vaccination HAI titers from other years were compared to 2015 values for the same individuals. Years with poor correlation (Pearson’s *r* < 0.5; for example, 2007, 2008, 2011 and 2012) were excluded from downstream analyses.

### Mice and immunization

Male C57BL/6J mice (6–10 weeks old) were obtained from the Jackson Laboratory and used for all experiments described in this study (*n* = 5 mice per experimental group). Mice were housed in the Stanford Animal Facility under specific-pathogen-free conditions, maintained on a 12-h light–dark cycle at a temperature of ~18–23 °C and 40–60% humidity. All animal procedures were reviewed and approved by the University Administrative Panel on Laboratory Animal Care (protocol no. 34513).

For immunization, each mouse received a total of 120 μl of intramuscular injections, administered as 60 μl into each hind leg (left and right caudal thigh muscles). The injection mixture contained 2023/24 IIV (Fluzone Quadrivalent) at a dose of 1 or 10 μg per mouse, combined with LNP-encapsulated mRNA encoding mouse cytokine adjuvants at 3 μg per mouse.

### Cytokine measurements

Cytokine levels were measured using Luminex-based multiplex liquid array assays at the Stanford HIMC. All samples were run in duplicate, and control beads (Radix Biosolutions) were included in every well. Depending on the year and platform version, the following kits were used:

- EMD Millipore Human 80-plex kits (these included three panels). Panel 1 was Milliplex HCYTA-60K-PX48. Panel 2 was Milliplex HCP2MAG-62K-PX23. Panel 3 included the Milliplex HSP1MAG-63K-06 and HADCYMAG-61K-03 (Resistin, Leptin and HGF) to generate a nine-plex). Samples were diluted 3× for Panels 1 and 2 or 10× for Panel 3, with 25 µl of sample used per well. After overnight incubation with antibody-linked magnetic beads at 4 °C with shaking, plates were washed twice with wash buffer in a BioTek ELx405 washer (BioTek Instruments). Cytokines were detected with biotinylated secondary antibodies and streptavidin–phycoerythrin (incubations of 1 h and 30 min, respectively, at 22 °C with shaking). Plates were washed again as above. The readout was performed on a Luminex FlexMap3D instrument, with a minimum bead count threshold of 50 per cytokine.
- EMD Millipore Magnetic Bead kits (62- or 63-plex): Similar protocol as above, with overnight sample incubation and FlexMap3D readout. Wells yielding bead counts <50 for a given analyte were flagged for quality control.
- Affymetrix/eBioscience Polystyrene Bead kits (37-, 50- or 51-plex): Samples were incubated on filter-bottom plates (22 °C followed by 4 °C), then washed and detected as per kit instructions. Plates were read on a Luminex 200 instrument, requiring at least 100 beads per cytokine for data inclusion.

### Antibody response quantification

HAI titers were measured from serum collected on day 0 (pre-vaccination) and day 28 (post-vaccination), as previously described^3^. Serially diluted sera (25 µl in PBS) were mixed with 25 µl of virus containing four HA units in V-bottom 96-well plates. After 15 min at 22 °C, 50 µl of 0.5% chicken red blood cells (cRBCs) were added, followed by a 1-h incubation. The HAI titer was defined as the reciprocal of the highest serum dilution that inhibited hemagglutination, indicated by a compact cRBC pellet. For each individual, the antibody response was defined as the geometric mean fold-rise in titer across vaccine strains, calculated as the ratio of the day 28 titer to the day 0 titer. Fold-rise values were log_2_-transformed to normalize distributions. To allow comparison across study years, titers were further adjusted using quantile normalization. Individuals with baseline titers ≥40 were optionally excluded in some analyses to minimize the effects of pre-existing immunity.

### Multi-cohort correlation and meta-analysis

Individuals were stratified into subpopulations based on age group, sex, vaccine dose (standard versus high), baseline HAI titer quantile, study cohort and influenza season. Each subpopulation (*n* ≥ 5) was analyzed using linear regression to determine the relationship between the standardized pre-vaccination cytokine level and the log_2_ antibody response. The resulting regression slope (with its standard error) was taken as the subgroup’s cytokine–response effect size. We then performed a meta-analysis to synthesize these effects across all subgroups, using the rmeta package in R to fit a random-effects model. This approach provided an overall estimate of the correlation between pre-vaccine cytokine levels and antibody response while accounting for between-group heterogeneity.

### Standardization of cytokine concentrations

We standardized cytokine abundances using *z*-score normalization, allowing us to plot them across different years (Extended Data Fig. 7). Specifically, we computed a *z*-score for each cytokine using the mean and standard deviation derived from a reference subset of relatively young individuals (<40 years old) across all years. We selected this younger group, assuming they have more stable immune profiles over time, providing a consistent baseline. This approach is analogous to previously published methods for longitudinal immune monitoring^34^.

### Disease correlation analysis

We investigated associations between baseline cytokine levels and subsequent disease diagnoses in older adults. Seniors (>65 years) from flu vaccine study no. 15 reported new disease diagnoses in annual medical surveys. For each disease of interest, we compared the pre-vaccination IFNβ levels in samples collected within 2 years before diagnosis (cases) with those from disease-free, age-matched individuals (controls). Only cytokine data from individuals >65 years were used in this analysis. Group differences in IFNβ were evaluated to identify any pre-diagnostic cytokine elevations.

### Spleen organoid culture and vaccination

Spleen tissues were retrieved from the Donor Network West (an authorized organ-processing organization). The procedure is approved by Stanford IRB (Exemption as donors are deceased). Spleen and tonsil organoid cultures were established using previously described methods^12^. Tissue was dissected into roughly 3–5 mm and manually disrupted into a single-cell suspension by processing through a 100-μm strainer with a 5-ml syringe plunger. Enzymatic dissociation was unnecessary and did not improve the response to LAIV from cryopreserved cells. Tissue debris was reduced by Ficoll density gradient separation, although this step was not required for tonsil organoid development. After washing with complete medium (RPMI with GlutaMAX (Thermo Fisher, 61870127), 10% FBS, 1× nonessential amino acids (Thermo Fisher, 11140050), 1× sodium pyruvate (Thermo Fisher, 11360070), 1× penicillin–streptomycin (Thermo Fisher, 15240062), 1× Normocin (InvivoGen) and 1× insulin/selenium/transferrin cocktail (Thermo Fisher, 41400045) (Gibco), cells were enumerated and frozen into aliquots in FBS + 10% dimethylsulfoxide (DMSO). Frozen cells were stored at −140 °C until use.

Aliquots were thawed into complete medium for culture of cryopreserved cells, enumerated and resuspended to 6 × 10^7^ cells per ml for larger cultures or 2 × 10^7^ cells per ml for smaller cultures. Cells were plated, 100 μl per well, into permeable (0.4-μm pore size) membranes (24-well size PTFE or polycarbonate membranes in standard 12-well plates or 96-well polycarbonate membrane plates with single-well receiver trays; Corning or Millipore), with the lower chamber consisting of complete medium (1 ml for 12-well plates and 200 μl for 96-well plates) supplemented with 1 μg ml^−1^ of recombinant human B cell-activating factor (BAFF; BioLegend, 559608). Adding a small amount of BAFF improved total B cell survival (and thus increased overall cell recovery) but was not a requirement for plasmablast differentiation or antibody secretion. Vaccination was performed using Fluzone (IIV, diluted 1:10,000) or FluMist (LAIV, diluted 1:2,000), dose-optimized for organoid stimulation.

### Cytokine and antibody detection in organoids

#### Cytokine quantification

Cytokine abundance in organoid culture supernatants was measured using a DNA-barcoded NULISA (Alamar Biosciences), capable of femtomolar-level detection. Supernatants were collected on day 3 post-stimulation for cytokine profiling. Each sample (25 µl) was analyzed on the NULISA Inflammation Panel targeting approximately 250 cytokines and chemokines, using the automated ARGO HT platform at the Stanford HIMC. For stimulation studies comparing IIV, LAIV, IFNβ and combinations thereof, culture conditions were standardized and cytokine abundance was reported relative to unstimulated controls matched by donor.

For influenza-A-specific IgG detection, to evaluate antigen-specific antibody responses, influenza HA-specific IgG was quantified in organoid supernatants on day 7 post-vaccination using a Luminex-based multiplex immunoassay. For the cytokine adjuvant screen, 19 recombinant human cytokines were co-administered with IIV at three concentrations (1, 10 or 100 ng ml^−1^, with adjusted ranges for IL-1β and IL-18), and influenza-specific IgG was measured relative to IIV-only controls. Organoids were generated from dissociated human spleen and tonsil tissue and maintained in a 96-well format for high-throughput screening. All measurements were performed in biological replicates (*n* = 5 donors), and fluorescence intensities were normalized and log-transformed to assess fold changes over background stimulation levels.

### Low-cell-input organoid culture

Organoids were established using low-input cell seeding to scale up culture throughput. Cells were seeded in ultra-low attachment 96-well plates at 1.6 × 10^5^ cells per well in 200 µl of culture medium. The medium was partially exchanged (30%) every 2 days. On day 7, culture supernatants were collected for antibody quantification as described above.

### NULISAseq inflammation panel

The NULISAseq inflammation panel (Alamar Biosciences), performed at the Stanford University HIMC, is a multiplexed proximity ligation assay targeting 250 inflammation-associated proteins. The assay was processed automatically in the ARGO HT system (Alamar BioSciences). Each sample (25 μl) of was loaded on the sample plate, along with three sample controls, four negative controls and three inter-plate controls. After completion of the automated run, next-generation sequencing (Illumina) was performed on the pooled library. Data were generated using Alamar Command Center and NULISA Analysis Software via normalization to internal controls and inter-plate controls. Raw counts were normalized to internal and inter-plate controls and then log_2_-transformed to yield normalized protein quantity values.

### Viral antibody assay

As previously described, a custom Luminex assay was created to detect antibody responses to SARS-CoV-2 and other viral antigens^35^. Antigens of interest were coupled to barcoded beads (Luminex Corporation) according to the manufacturer’s instructions. Supernatant samples were run undiluted, with 25 μl of sample added to the assay plate containing the antigen-coupled bead mixture and incubated for 2 h at 22 °C or overnight at 4 °C, shaking on an orbital shaker. Samples were then washed and 25 μl of secondary goat-anti human IgG (Fc fragment) coupled to phycoerythrin (anti-IgG, NC9822979, Jackson ImmunoResearch) was added. After incubation and shaking for 30 min at 22 °C, a second wash was performed before adding 130 μl Reading Buffer (Luminex). Samples were read on a Luminex Flex 3D instrument with a lower bound of 50 beads per target antigen. This assay was performed by the Stanford University HIMC and is presented as median fluorescence intensity.

### RNA vaccine synthesis protocol

DNA sequences for vaccines were codon-optimized using an in-house algorithm and synthesized by Synbio Technologies. The 3′ and 5′ untranslated regions (UTRs) were taken from the commercial Pfizer vaccine^36^. Gene sequences were PCR amplified and cloned into a backbone containing 3′ and 5′ UTRs (NEBuilder, NEB). Then, constructs were amplified, linearized with a T7 promoter 5′ overhang and polyadenylated via a two-step PCR. PCR Steps were performed using 0.5 µM of each primer, 1–2 ng template per 50-µl reaction and Platinum SuperFi II polymerase (Thermo Fisher). PCRs were cleaned up via standard protocol (QIAGEN). In vitro transcription of purified PCR products was performed using the T7 mScript Standard mRNA production kit (Cellscript), with N1-methylpseudouridine (Trilink Biotech) instead of uracil. Then, RNA was capped using the ScriptCap Cap 1 Capping System (Cellscript). RNA was purified via the Monarch RNA Cleanup kit. IVT RNA was stored at −80 °C and was used to generate LNPs contained the same lipid components as the commercial Pfizer vaccine^36^, including ALC-0315 (5.41 mg ml^−1^, 7.06 mM), ALC-0159 (0.629 mg ml^−1^, 0.26 mM), cholesterol (2.52 mg ml^−1^, 6.52 mM) and 1,2-distearoyl-sn-glycero-3-phosphocholine (DSPC) (1.13 mg ml^−1^, 1.43 mM), prepared from stock solutions (50, 10, 10 and 5 mg ml^−1^, respectively) in ethanol (150.9 µl 100% ethanol and 200 µl 50% ethanol) to a final volume of 1 ml.

RNA was thawed and diluted to final concentrations of 141 ng µl^−1^ RNA, 25 mM acetate and 200 mM NaCl, pH 4. Then, RNA and lipid solutions underwent microfluidic mixing at a 3:1 volume ratio of RNA to lipid. Solutions were loaded into 1-ml syringes, as larger plastic syringes flex under pressure and are unsuitable for mixing. It is essential that the syringes contain almost no air pockets, or mixing will not occur reliably. For small batches, syringes can be pre-filled with buffer, and a small air pocket can be used to separate RNA from RNA buffer along the length of the tubing. For large batches, 1-ml syringes were filled. Solutions flowed from the syringe to the microfluidic mixer via Cole Parmer Masterflex Microbore Transfer Tubing (Tygon ND-100-80, 0.020′ internal diameter (ID) × 0.060′ outer diameter (OD)), and into the device via custom tubing adapters (0.025′ OD × 0.013′ ID, 0.5′ length type 304 stainless steel, New England Small Tube). The overall flow rate through the device was 500 μL per minute, controlled via a syringe pump (New Era Pump Systems Inc. NE-4000).

The output solution was dialyzed against PBS to remove ethanol (Pierce microdialysis devices, 0.3-ml capacity, 3.5 kDa MWCO), typically diluting the final vaccine to 30 µg RNA per 400 µl. If the LNPs were to be frozen, the vaccine was further diluted by adding 1:2 volume of sterile-filtered 60% sucrose in PBS (final sucrose of 20% w/v). LNPs were then flash frozen in liquid nitrogen and placed in LN2 storage. The microfluidic mixer used for LNP synthesis was fabricated using standard photolithography; the corresponding photomask design is available upon request. Photomasks were ordered from Artnet Pro. The flow channel layer was fabricated to a height of roughly 63 μm and the herringbone layer was manufactured to a height of approximately 21 μm. The photomask was drafted in-house, but the basis for this mixer was previously published^37^.

### ELISpot analysis

Bone-marrow ELISpot assays were performed on day 434 after the first immunization, as previously described^38^. In brief, multiscreen 96-well plates with Immobilon-P membrane (Millipore, MAIPS4510) were coated overnight at 4 °C with 50 μl per well of Fluzone Quadrivalent 2023/24 seasonal influenza virus vaccine at a final concentration of 2 μg ml^−1^ in PBS. Plates were washed with PBS and blocked with RPMI 1640/10% FBS medium at 37 °C for 2 h. Single-cell suspensions were prepared from the bone marrow of femurs and tibias, followed by red blood cell lysis using ACK lysis buffer. Cells were then plated onto antigen-coated wells and incubated for 15 h at 37 °C. After washing, an HRP-conjugated anti-mouse IgG antibody (SouthernBiotech, 1031-05, 1:2,500 dilution) was added, and antigen-specific responses were visualized using an AEC substrate detection kit (BD Biosciences, 551951). ELISpot plates were scanned using ImmunoSpot S6 Universal M2 analyzer and analyzed using an automated ELISpot counting system (Cellular Technology). The number of spots per million cells was calculated.

### Fluorescence imaging

To assess the distribution of LNP-encapsulated mRNA transcripts, mice were intramuscularly immunized with PBS or LNP-encapsulated mRNA encoding GFP (3 μg per mouse) as described. Draining lymph nodes and hind limb muscles were collected at 24 h post-immunization. Whole-tissue fluorescence was then measured using a Largo spectral imaging system with an excitation wavelength of 465 nm and an emission wavelength of 510 nm. The data were analyzed using Aura imaging software (Spectral Instruments Imaging) and values represent the integrated fluorescence intensity.

### Statistics and reproducibility

No statistical method was used to predetermine sample size. Human cohort analyses were based on available samples from previously conducted studies as described above. No data were excluded from human studies unless explicitly stated in the Methods (for example, exclusion of years with poor inter-year correlation as described above). The experiments were not randomized. For organoid experiments, all conditions were tested across all donor samples, and therefore randomization was not applicable. For mouse studies, animals were assigned to experimental groups without randomization. No animals were excluded from the analyses. Investigators were not blinded to allocation during experiments or outcome assessment. Statistical analyses were performed using R (R Foundation for Statistical Computing). Data distribution was assumed to be normal but this was not formally tested.

### Reporting summary

Further information on research design is available in the Nature Portfolio Reporting Summary linked to this article.

## Online content

Any methods, additional references, Nature Portfolio reporting summaries, source data, extended data, supplementary information, acknowledgements, peer review information; details of author contributions and competing interests; and statements of data and code availability are available at 10.1038/s41590-026-02547-x.

## Supplementary information

Supplementary Information Supplementary Tables 1 and 2.

Reporting Summary

Peer Review File

## Source data

Source Data Fig. 1 Source data for cytokine levels and antibody responses shown in Fig. 1b,c.

Source Data Fig. 2 Source data for antibody response analyses shown in Fig. 2.

Source Data Fig. 3 Source data for cytokine–response correlations shown in Fig. 3b.

Source Data Fig. 4 Source data for organoid cytokine and antibody responses in Fig. 4b–e.

Source Data Fig. 6 Source data for in vivo antibody responses shown in Fig. 6.

Source Data Extended Data Fig.1 Source data for Extended Data Fig. 1.

Source Data Extended Data Fig. 5 Source data for Extended Data Fig. 5b.

Source Data Extended Data Fig. 7 Source data for Extended Data Fig. 7a,b,e.

## Data Availability

All data supporting the findings of this study are provided as Source Data files with this paper. Source data are provided with this paper.

## Code availability

The analysis code used in this study has been deposited in Zenodo at 10.5281/zenodo.20125292 (ref. ^39^).
