# Supplementary material for: Human vaccine responses regulated by parallel cytokine pathways
Source: Nat Immunol. 2026 Jun 12;27(8):1643–52. doi: 10.1038/s41590-026-02547-x (PMC13414556; doi:10.1038/s41590-026-02547-x)
Supplement: Supplementary file 1 — Supplementary Tables 1 and 2. [file 41590_2026_2547_MOESM1_ESM.pdf]

nature immunology

Supplementary information

https://doi.org/10.1038/s41590-026-02547-x

# Human vaccine responses regulated by parallel cytokine pathways

In the format provided by the authors and unedited

**Table S1: Literature Summary of Cytokine Adjuvant Effect (IgG production) in Mouse Studies**

| Cytokine Tested | Cytokine Format | Antigen Species | Antigen | Delivery Route | Effect on IgG | Ref |
|---|---|---|---|---|---|---|
| IFNα/β | Protein | None - Model Antigen | Chicken Gamma Globulin | SC | Pos | 8 |
| IFNα/β | Protein | Influenza | Influenza Vaccine | IM | Pos | 9 |
| IFNγ | Protein | HIV | gp-120 | IP | Pos | 10 |
| IFNγ | Protein | Influenza | Influenza Vaccine | IP | Pos | 11 |
| IL-10 | DNA | HIV | Gag/pol; ENV | IM | Pos | 12 |
| IL-12 | Protein | Influenza | H1, N1 | IN | Pos | 13 |
| IL-15 | Protein | *Staphylococcus aureus* | STEBVax toxoid | IM | Pos | 14 |
| IL-17 | Protein | BoHV-5 | BoHV-5 gD | IM | Pos | 15 |
| IL-18 | DNA | HIV | Gag/pol; ENV | IM | Pos | 12 |
| IL-18 | DNA | *Schistosoma japonicum* | GST | IM | None | 16 |
| IL-18 | Protein | Influenza | HA | IN | Pos | 17 |
| IL-18 | RNA | Influenza | Split Influenza Vaccine | IM | Pos | 18 |
| IL-1α/β | Protein | None - Model Protein | BSA | IP | Pos | 19 |
| IL-1α/β | Protein | None - Model Antigen | Ovalbumin | MOP | Pos | 20 |
| IL-1α/β | Protein | Influenza | HA | IN | Pos | 17 |
| IL-1α/β | Protein | None - Model Antigen | Ovalbumin or TT | IN | Pos | 21 |
| IL-1β | Protein | *Clostridium difficile* | Flagellar Cap FliD Protein | OG | None | 22 |
| IL-2 | DNA | HIV | Gag/pol; ENV | IM | Pos | 12 |
| IL-2 | DNA | Influenza | Influenza Vaccine | SC | Pos | 23 |
| IL-21 | DNA | HIV | ENV Peptide | IM | Neg | 24 |
| IL-21 | DNA | HBV | HBV Envelope | IM | None | 25 |
| IL-21 | DNA | None - Tumor Model | SCCVII | IV | Pos | 26 |
| IL-21 | DNA | None- Tumor Model | GD2 | IM | Pos | 27 |
| IL-21 | DNA | HIV | Gag + MVA | IM | None | 28 |
| IL-21 | Cell | TB | ESAT-6 | SC | None | 29 |
| IL-4 | DNA | HIV | Gag/pol; ENV | IM | Pos | 12 |
| IL-4 | DNA | Influenza | Influenza Vaccine | SC | Pos | 23 |
| IL-5 | DNA | HIV | Gag/pol; ENV | IM | Pos | 12 |
| IL-9 | DNA | FMDV | VP1 | IM | Pos | 30 |
| LTA | DNA | HIV | Gag/pol; ENV | IM | Pos | 12 |
| TNF | DNA | HIV | Gag/pol; ENV | IM | Pos | 12 |

Abbreviations: Subcutaneous (SC), Intramuscular (IM), Intraperitoneal (IP), Intranasal (IN) Mini Osmotic Pump (MOP), Intravenous (IV). Effect on IgG determined by author description or based on average IgG fold change or IgG O.D. change values when provided Pos (>1X with strong trend or significance), None (1X, inconsistent, or not significant), Neg (<1X).

## Supplemental References


1 Brodin, P. *et al.* Variation in the Human Immune System Is Largely Driven by Non-Heritable Influences. *Cell* **160**, 37-47 (2015). https://doi.org/10.1016/j.cell.2014.12.020

2 Alpert, A. *et al.* A clinically meaningful metric of immune age derived from high-dimensional longitudinal monitoring. *Nat Med* **25**, 487-+ (2019). https://doi.org/10.1038/s41591-019-0381-y

3 Wagar, L. E. *et al.* Modeling human adaptive immune responses with tonsil organoids. *Nat Med* **27**, 125-135 (2021). https://doi.org/10.1038/s41591-020-01145-0

4 Rosenberg-Hasson, Y. *et al.* Relationship of Heterologous Virus Responses and Outcomes in Hospitalized COVID-19 Patients. *Journal of Immunology* **211**, 1224-1231 (2023). https://doi.org/10.4049/jimmunol.2300391

5 WHO. Vol. 34 *WHO Drug Information* 271-384 (World Health Organization,, Geneva, Switzerland, 2020).

6 Chen, D. L. *et al.* Rapid Discovery of Potent siRNA-Containing Lipid Nanoparticles Enabled by Controlled Microfluidic Formulation. *J Am Chem Soc* **134**, 6948-6951 (2012). https://doi.org/10.1021/ja301621z

7 Yin, Q. *et al.* A TLR7-nanoparticle adjuvant promotes a broad immune response against heterologous strains of influenza and SARS-CoV-2. *Nat Mater* **22**, 380-390 (2023). https://doi.org/10.1038/s41563-022-01464-2

8 Le Bon, A. *et al.* Type I interferons potently enhance humoral immunity and can promote isotype switching by stimulating dendritic cells in vivo. *Immunity* **14**, 461-470 (2001). https://doi.org/Doi 10.1016/S1074-7613(01)00126-1

9 Proietti, E. *et al.* Type I IFN as a natural adjuvant for a protective immune response: Lessons from the influenza vaccine model. *Journal of Immunology* **169**, 375-383 (2002). https://doi.org/DOI 10.4049/jimmunol.169.1.375

10 McCormick, A. L., Thomas, M. S. & Heath, A. W. Immunization with an interferon-γ-gp120 fusion protein induces enhanced immune responses to human immunodeficiency virus gp120. *J Infect Dis* **184**, 1423-1430 (2001). https://doi.org/Doi 10.1086/324371

11 van Slooten, M. L. *et al.* Immunoadjuvant activity of interferon-γ-liposomes co-administered with influenza vaccines. *Bba-Mol Cell Biol L* **1531**, 99-110 (2001). https://doi.org/10.1016/S1388-1981(01)00092-0

12 Kim, J. J. *et al.* Modulation of amplitude and direction of immune responses by co-administration of cytokine gene expression cassettes with DNA immunogens. *Eur J Immunol* **28**, 1089-1103 (1998). https://doi.org/10.1002/(SICI)1521-4141(199803)28:03<1089::AID-IMMU1089>3.0.CO;2-L

13 Arulanandam, B. P., O'Toole, M. & Metzger, D. W. Intranasal interleukin-12 is a powerful adjuvant for protective mucosal immunity. *J Infect Dis* **180**, 940-949 (1999). https://doi.org/Doi 10.1086/314996

14 Saikh, K. U., Kissner, T. L., Nystrom, S., Ruthel, G. & Ulrich, R. G. Interleukin-15 increases vaccine efficacy through a mechanism linked to dendritic cell maturation and enhanced antibody titers. *Clin Vaccine Immunol* **15**, 131-137 (2008). https://doi.org/10.1128/Cvi.00320-07

15 Gonçalves, V. S. *et al.* Recombinant bovine IL17A acts as an adjuvant for bovine herpesvirus vaccine. *Res Vet Sci* **136**, 185-191 (2021). https://doi.org/10.1016/j.rvsc.2021.02.014

16 Wei, F. *et al.* Enhancement by IL-18 of the protective effect of a 26 kDa GST plasmid DNA vaccine in mice. *Vaccine* **26**, 4145-4149 (2008). https://doi.org/10.1016/j.vaccine.2008.05.034

17 Kayamuro, H. *et al.* Interleukin-1 Family Cytokines as Mucosal Vaccine Adjuvants for Induction of Protective Immunity against Influenza Virus. *J Virol* **84**, 12703-12712 (2010). https://doi.org/10.1128/Jvi.01182-10

18 Lee, Y. S. *et al.* Analysis of the Immunostimulatory Effects of Cytokine-Expressing Internal Ribosome Entry Site-Based RNA Adjuvants and Their Applications. *J Infect Dis* **229**, 1408-1418 (2024). https://doi.org/10.1093/infdis/jiad392

19 Staruch, M. J. & Wood, D. D. The adjuvanticity of interleukin 1 in vivo. *J Immunol* **130**, 2191-2194 (1983).

20 Ben-Sasson, S. Z. *et al.* IL-1 acts directly on CD4 T cells to enhance their antigen-driven expansion and differentiation. *P Natl Acad Sci USA* **106**, 7119-7124 (2009). https://doi.org/10.1073/pnas.0902745106

21 Staats, H. F. & Ennis, F. A. IL-1 is an effective adjuvant for mucosal and systemic immune responses when coadministered with protein immunogens. *Journal of Immunology* **162**, 6141-6147 (1999).
22 Potocki, W. *et al.* IL-1 Fragment Modulates Immune Response Elicited by Recombinant Spores Presenting an Antigen/Adjuvant Chimeric Protein. *Mol Biotechnol* **60**, 810-819 (2018). https://doi.org/10.1007/s12033-018-0117-0
23 Herbert, A. S., Heffron, L., Sundick, R. & Roberts, P. C. Incorporation of membrane-bound, mammalian-derived immunomodulatory proteins into influenza whole virus vaccines boosts immunogenicity and protection against lethal challenge. *Virol J* **6** (2009). https://doi.org/10.1186/1743-422X-6-42
24 Feng, C. C. *et al.* Interleukin-21 Inhibits Humoral Response to an HIV DNA Vaccine by Enhancing Bcl-6 and Pax-5 Expression. *Viral Immunol* **25**, 131-140 (2012). https://doi.org/10.1089/vim.2011.0073
25 Zhang, Y. *et al.* A Fusion DNA Vaccine Encoding Middle Version of HBV Envelope Protein Fused to Interleukin-21 Did Not Enhance HBV-Specific Immune Response in Mice. *Viral Immunol* **27**, 430-437 (2014). https://doi.org/10.1089/vim.2014.0051
26 Nakano, H. *et al.* Interleukin-21 triggers both cellular and humoral immune responses leading to therapeutic antitumor effects against head and neck squamous cell carcinoma. *J Gene Med* **8**, 90-99 (2006). https://doi.org/10.1002/jgm.817
27 Kowalczyk, A. *et al.* Induction of protective immune responses against NXS2 neuroblastoma challenge in mice by immunotherapy with GD2 mimotope vaccine and IL-15 and IL-21 gene delivery. *Cancer Immunol Immun* **56**, 1443-1458 (2007). https://doi.org/10.1007/s00262-007-0289-0
28 Fan, W. J., Wan, Y. M. & Li, Q. S. Interleukin-21 enhances the antibody avidity elicited by DNA prime and MVA boost vaccine. *Cytokine* **125** (2020). https://doi.org/ARTN15481410.1016/j.cyto.2019.154814
29 He, X. F. *et al.* Antitumor efficacy of viable tumor vaccine modified by heterogenetic ESAT-6 antigen and cytokine IL-21 in melanomatous mouse. *Immunol Res* **52**, 240-249 (2012). https://doi.org/10.1007/s12026-012-8332-4
30 Zou, Q. *et al.* Increasing a Robust Antigen-Specific Cytotoxic T Lymphocyte Response by FMDV DNA Vaccination with IL-9 Expressing Construct. *J Biomed Biotechnol* (2010). https://doi.org/Artn 56235610.1155/2010/562356

**Table S2: Demographic characteristics of human organoid donors**

| Sample ID | Age | Gender | Ethnicity | Cause of death |
|---|---|---|---|---|
| Spleen 44 | 59 | M | White | Head trauma/Blunt injury/Accident |
| Spleen 53 | 70 | F | White | Anoxia/Cardiovascular |
| Spleen 54 | 62 | F | Asian | Cerebrovascular/Stroke/Intracranial hemorrhage |
| Tonsil 55 | 52 | M | White | N/A |
| Tonsil 159 | 37 | M | White | N/A |
| Tonsil 161 | 29 | M | Hispanic/Latino | N/A |
