# Supplementary material for: Human vaccine responses regulated by parallel cytokine pathways
Source: Nat Immunol. 2026 Jun 12;27(8):1643–52. doi: 10.1038/s41590-026-02547-x (PMC13414556; doi:10.1038/s41590-026-02547-x)
Supplement: Supplementary file 2 — Reporting Summary [file 41590_2026_2547_MOESM2_ESM.pdf]

nature portfolio

Corresponding author(s): Bill Chen, Mark Davis, Yueh-hsiu Chien

Last updated by author(s): 4/27/2026

Reporting Summary

Nature Portfolio wishes to improve the reproducibility of the work that we publish. This form provides structure for consistency and transparency in reporting. For further information on Nature Portfolio policies, see our Editorial Policies and the Editorial Policy Checklist.

Statistics

For all statistical analyses, confirm that the following items are present in the figure legend, table legend, main text, or Methods section.

n/a	Confirmed	
☐	☒	The exact sample size (*n*) for each experimental group/condition, given as a discrete number and unit of measurement
☐	☒	A statement on whether measurements were taken from distinct samples or whether the same sample was measured repeatedly
☐	☒	The statistical test(s) used AND whether they are one- or two-sided<br>*Only common tests should be described solely by name; describe more complex techniques in the Methods section.*
☒	☐	A description of all covariates tested
☐	☒	A description of any assumptions or corrections, such as tests of normality and adjustment for multiple comparisons
☐	☒	A full description of the statistical parameters including central tendency (e.g. means) or other basic estimates (e.g. regression coefficient) AND variation (e.g. standard deviation) or associated estimates of uncertainty (e.g. confidence intervals)
☐	☒	For null hypothesis testing, the test statistic (e.g. *F*, *t*, *r*) with confidence intervals, effect sizes, degrees of freedom and *P* value noted<br>*Give P values as exact values whenever suitable.*
☒	☐	For Bayesian analysis, information on the choice of priors and Markov chain Monte Carlo settings
☒	☐	For hierarchical and complex designs, identification of the appropriate level for tests and full reporting of outcomes
☐	☒	Estimates of effect sizes (e.g. Cohen's *d*, Pearson's *r*), indicating how they were calculated

Our web collection on statistics for biologists contains articles on many of the points above.

Software and code

Policy information about availability of computer code

Data collection	Data were collected using the standard software associated with each instrument.
Data analysis	Data analysis used R (including the rmeta package). Figures and schematic elements were assembled using Adobe Illustrator, and Adobe InDesign, as well as tools from the Microsoft Office suite.

For manuscripts utilizing custom algorithms or software that are central to the research but not yet described in published literature, software must be made available to editors and reviewers. We strongly encourage code deposition in a community repository (e.g. GitHub). See the Nature Portfolio guidelines for submitting code & software for further information.

Data

Policy information about availability of data

All manuscripts must include a data availability statement. This statement should provide the following information, where applicable:

- Accession codes, unique identifiers, or web links for publicly available datasets
- A description of any restrictions on data availability
- For clinical datasets or third party data, please ensure that the statement adheres to our policy

Data Availability
All data supporting the findings of this study are provided as Source Data files with this paper.

Code Availability
The analysis code used in this study has been deposited in Zenodo and is available at https://doi.org/10.5281/zenodo.20125292.

Research involving human participants, their data, or biological material

Policy information about studies with human participants or human data. See also policy information about sex, gender (identity/presentation), and sexual orientation and race, ethnicity and racism.

Reporting on sex and gender	Both male and female participants were included in the influenza vaccine cohort, which had an overall sex distribution of approximately 58% female. Sex was included as a covariate in subgroup analyses.For human immune organoid experiments, both male and female donors were included. Sex was recorded for all donors but was not used as a variable in downstream analyses due to limited sample size.
Reporting on race, ethnicity, or other socially relevant groupings	Race and ethnicity information was not available for the influenza vaccine cohort and was not included in the analysis.For organoid donors, ethnicity information was available for all donors and is reported in Supplementary Table S2 but was not used in analyses due to limited sample size.
Population characteristics	Participants were drawn from influenza vaccine cohorts (n = 581), with ages ranging from 2 to 90+ years and an overall sex distribution of 58% female.Human immune organoid experiments were performed using primary tissues from independent donors, including n = 5 donors (3 tonsil and 2 spleen donors) for cytokine screening experiments and n = 3 spleen donors for cytokine profiling experiments.For organoid donors, demographic information including age (29–70 years), sex, and ethnicity was available for all donors and is provided in Supplementary Table S2. Cause of death information (for deceased donors) was not available for all samples and is provided where available in Supplementary Table S2. Tissues were obtained as de-identified surgical discards or through deceased-donor procurement processes, and no demographic or behavioral selection criteria were applied.
Recruitment	Participants were enrolled in influenza vaccine studies conducted by the Stanford Human Immune Monitoring Center (HIMC) across multiple seasons, as described in the Methods section.No human participants were recruited for the organoid studies. Organ donor tissues were obtained through standard procurement processes (Donor Network West).No direct interaction with donors occurred.
Ethics oversight	Human cohort studies were approved by the Stanford University Administrative Panels on Human Subjects in Medical Research (IRB protocol #62436). Use of deceased donor spleen tissue was exempt under Stanford IRB guidelines via Donor Network West. Tonsil tissues were obtained as de-identified surgical discards under institutional approval.

Note that full information on the approval of the study protocol must also be provided in the manuscript.

Field-specific reporting

Please select the one below that is the best fit for your research. If you are not sure, read the appropriate sections before making your selection.

☒ Life sciences ☐ Behavioural & social sciences ☐ Ecological, evolutionary & environmental sciences

For a reference copy of the document with all sections, see nature.com/documents/nr-reporting-summary-flat.pdf

Life sciences study design

All studies must disclose on these points even when the disclosure is negative.

Sample size	Sample sizes were determined by the availability of human donor tissues and by the fixed group sizes in previously completed influenza vaccine cohort studies (n-values shown in figure legends). For mouse experiments, group sizes of five animals per condition were chosen based on prior work showing robust detection of antibody responses.
Data exclusions	No data were excluded from the analyses.
Replication	All experimental measurements were performed in biological replicates as indicated by donor or mouse n-values, and key findings were independently reproduced across multiple donors or experiments.
Randomization	Mouse experiments involved allocation of animals into groups without formal randomization and no covariates required controlled allocation. Randomization was not relevant for human donor tissues or previously collected cohort data.
Blinding	Blinding was not performed because group identities were required for sample processing and analysis; data were collected and analyzed using standardized, quantitative assays that minimize investigator bias.

Reporting for specific materials, systems and methods

We require information from authors about some types of materials, experimental systems and methods used in many studies. Here, indicate whether each material, system or method listed is relevant to your study. If you are not sure if a list item applies to your research, read the appropriate section before selecting a response.

Materials & experimental systems

n/a	Involved in the study
☒	☐ Antibodies
☒	☐ Eukaryotic cell lines
☒	☐ Palaeontology and archaeology
☐	☒ Animals and other organisms
☐	☒ Clinical data
☒	☐ Dual use research of concern
☒	☐ Plants

Methods

n/a	Involved in the study
☒	☐ ChIP-seq
☒	☐ Flow cytometry
☒	☐ MRI-based neuroimaging

Animals and other research organisms

Policy information about studies involving animals; ARRIVE guidelines recommended for reporting animal research, and Sex and Gender in Research

Laboratory animals	Male C57BL/6J mice (6–10 weeks old) were used and are described in the Methods, including housing and dosing details.
Wild animals	This study did not involve wild animals.
Reporting on sex	Male mice were used; sex-based analyses were not performed.
Field-collected samples	This study did not involve field-collected samples.
Ethics oversight	All animal procedures were approved by the Stanford University Administrative Panel on Laboratory Animal Care (APLAC; protocol no. 34513).

Note that full information on the approval of the study protocol must also be provided in the manuscript.

Clinical data

Policy information about clinical studies

All manuscripts should comply with the ICMJE guidelines for publication of clinical research and a completed CONSORT checklist must be included with all submissions.

Clinical trial registration	Not applicable; this study did not involve a clinical trial.
Study protocol	Not applicable; no interventional study involving human participants was conducted.
Data collection	Human cohort cytokine and antibody data were obtained from previously completed IRB-approved influenza vaccine studies at the Stanford Human Immune Monitoring Center.
Outcomes	Not applicable; no clinical trial outcomes were defined.

Plants

Seed stocks	n/a - the form is having an error when you click involved in the study the plants areas disappear, when you select n/a the boxes appear.
Novel plant genotypes	n/a - the form is having an error when you click involved in the study the plants areas disappear, when you select n/a the boxes appear.
Authentication	n/a - the form is having an error when you click involved in the study the plants areas disappear, when you select n/a the boxes appear.
