# Supplementary material for: Human vaccine responses regulated by parallel cytokine pathways
Source: Nat Immunol. 2026 Jun 12;27(8):1643–52. doi: 10.1038/s41590-026-02547-x (PMC13414556; doi:10.1038/s41590-026-02547-x)
Supplement: Supplementary file 3 — Peer Review File [file 41590_2026_2547_MOESM3_ESM.pdf]

nature portfolio

Peer Review File

Human Vaccine Responses Regulated by Parallel Cytokine Pathways

Corresponding Author: Dr Guangbo (Bill) Chen

This file contains all editorial decision letters in order by version, followed by all author rebuttals in order by version.

Decision Letter:

14th Jan 2026

Dear Bill,

Thank you for providing a point-by-point response to the concerns posed by referee #2 on your manuscript entitled "Human Vaccine Responses Regulated by Parallel Cytokine Pathways". As noted in my previous e-mail message, while they find your work of interest, this referee requested clarifications on several important points that would raise the impact of the current study. We are very interested in the possibility of publishing your study in Nature Immunology, but would like to consider your response to these concerns in the form of a revised manuscript before we make a final decision on publication.

We invite you to submit a substantially revised manuscript, however please bear in mind that we will be reluctant to approach the referees again in the absence of major revisions.

Specifically, as noted in your response, the revision should include new experiments to extend the in vivo studies to include later time points to strengthen the conclusions for antibody persistence, as well as to perform multiple-comparison corrections in the statistical analysis of the datasets associated with this study.

Please include the additional textual clarifications as indicated in your response letter.

When you revise your manuscript, please take into account all reviewer and editor comments, please highlight all changes in the manuscript text file in Microsoft Word format.

We are committed to providing a fair and constructive peer-review process. Do not hesitate to contact us if there are specific requests from the reviewers that you believe are technically impossible or unlikely to yield a meaningful outcome.

When revising your manuscript:

* Include a “Response to referees” document detailing, point-by-point, how you addressed each referee comment. If no action was taken to address a point, you must provide a compelling argument. This response will be sent back to the referees along with the revised manuscript.

* If you have not done so already please begin to revise your manuscript so that it conforms to our Article format instructions at http://www.nature.com/ni/authors/index.html. Refer also to any guidelines provided in this letter.

* Please include a revised version of any required reporting checklist. It will be available to referees to aid in their evaluation of the manuscript goes back for peer review. They are available here:

Reporting summary:
https://www.nature.com/documents/nr-reporting-summary.pdf

When submitting the revised version of your manuscript, please pay close attention to our href="https://www.nature.com/nature-portfolio/editorial-policies/image-integrity">Digital Image Integrity Guidelines. and to the following points below:

-- that unprocessed scans are clearly labelled and match the gels and western blots presented in figures.
-- that control panels for gels and western blots are appropriately described as loading on sample processing controls
-- all images in the paper are checked for duplication of panels and for splicing of gel lanes.

Please note, Extended Data figures and tables are online-only (appearing in the online PDF and full-text HTML version of the paper), peer-reviewed display items that provide essential background to the Article but are not included in the printed version of the paper due to space constraints or being of interest only to a few specialists. A maximum of ten Extended Data display items (figures and tables) is typically permitted. When re-submitting your manuscript, please ensure that any supplementary figures and tables that are more critical to the manuscript's conclusions are converted to Extended data to increase these data's visibility.

Finally, please ensure that you retain unprocessed data and metadata files after publication, ideally archiving data in perpetuity, as these may be requested during the peer review and production process or after publication if any issues arise.

Please use the link below to submit your revised manuscript and related files:

Link Redacted

Note: This URL links to your confidential home page and associated information about manuscripts you may have submitted, or that you are reviewing for us. If you wish to forward this email to co-authors, please delete the link to your homepage.

We hope to receive your revised manuscript within two weeks. If you cannot send it within this time, please let us know. We will be happy to consider your revision so long as nothing similar has been accepted for publication at Nature Immunology or published elsewhere.

Please do not hesitate to contact me if you have any questions or would like to discuss these revisions further.

Nature Immunology is committed to improving transparency in authorship. As part of our efforts in this direction, we are now requesting that all authors identified as 'corresponding author' on published papers create and link their Open Researcher and Contributor Identifier (ORCID) with their account on the Manuscript Tracking System (MTS), prior to acceptance. ORCID helps the scientific community achieve unambiguous attribution of all scholarly contributions. You can create and link your ORCID from the home page of the MTS by clicking on 'Modify my Springer Nature account'. For more information please visit please visit www.springernature.com/orcid.

We look forward to seeing the revised manuscript and thank you for the opportunity to review your work.

Best wishes for the New Year,

Laurie

Laurie A. Dempsey, Ph.D.
Senior Editor
Nature Immunology
l.dempsey@us.nature.com
ORCID: 0000-0002-3304-796X

Version 0:

Reviewer comments:

Reviewer #1

(Remarks to the Author)
The manuscript identifies the factors influencing the human antibody response against influenza vaccines.
The initial analysis examined in vivo human responses to the inactivated influenza vaccine (IIV). By pooling data from 581 individuals vaccinated across five influenza seasons, the authors correlated antibody responses with age and serum cytokine levels. Older age was associated with significantly reduced antibody responses, particularly in individuals with low pre-vaccination antibody titers. Among 66 measured factors, higher concentrations of IL-18, IFN-β, and the chemokine GRO-α were associated with stronger antibody responses.

To validate these findings under controlled and reproducible conditions, the authors developed a human immune organoid system to assess the effects of cytokines identified in the human studies. Using immune organoids generated from three tonsils and two spleens, they evaluated responses to IIV and the modulatory role of cytokines. All type I interferons enhanced antibody production, as did IL-21 and IL-12, whereas IL-18 and IFN-γ did not show a direct effect.

Further experiments revealed that IIV exposure induced type II interferon (IFN-γ) but not type I interferon responses in the organoids. In contrast, the live attenuated influenza vaccine (LAIV) induced robust type I and type III interferon responses. IFN-β stimulation in organoids recapitulated the cytokine profile induced by LAIV. Thus, IFN-β mimics the superior immunogenicity of live vaccines.
LAIV also induced IL-12 and IL-21, which further enhanced antibody responses.

The main findings of this important study are twofold. First, IFN-β acts as a natural adjuvant and recapitulates the cytokine response induced by the live attenuated influenza vaccine (LAIV). Second, in mice, combining the inactivated influenza vaccine (IIV) with mRNA–lipid nanoparticles (mRNA-LNPs) leads to the generation of high levels of strain-specific and cross-reactive antibodies, with both IL-21 and IFN-β mRNA-LNPs inducing higher and more durable antibody responses.

The elegance and scientific value of the work lie in its comprehensive, iterative experimental approach. Observations made in clinical samples are validated in human immune organoid models and subsequently confirmed in vivo. Finally, mouse models are used to both corroborate and extend these findings, establishing a potential translational vaccination strategy.

Reviewer #2

(Remarks to the Author)
In this report, the authors evaluated the association between pre-vaccination serum cytokines and the responsiveness to and immunogenicity of the inactivated influenza vaccine (IIV) in people, using data from 4 cohorts collected between 2009 and 2015. Consistent with previous reports, their analysis revealed that individuals with low pre-vaccination influenza antibody levels showed a greater post-vaccination boost in vaccine response, and this boost was significantly greater in people younger than 65 years than in those older than 65 years. Focusing on this Pre-Vax FluAb Low group, the authors performed a meta-analysis of the association between 66 different serum cytokines on the day of boost (D0) and vaccine potency (the ratio of Ab response at D28 vs D0 post-vaccination) and found three cytokines/chemokines, IL-18, GROa, and IFNb, showing the most significant positive association. To determine whether these cytokines were responsible for the improved vaccine potency, they used a human tonsil/spleen organoid model. They studied the influence of these cytokines on IIV vaccine-induced generation of flu-specific antibody. The results confirmed a role for IFNb in enhancing the antibody response but not IL-18. In addition, the results showed that other cytokines, such as IL-21, IL-10, and IL-12, enhanced the generation of the antibody response. To test the adjuvant effects of IFNb and IL-21 in vivo, the authors vaccinated mice with IIV in the presence or absence of mRNA-LNPs expressing IL-21 or IFNb. They found that IL-21 significantly enhanced the magnitude of the antibody response against homologous and heterologous HAs. Overall, the authors conclude that pre-existing serum cytokines, such as IFNb, act as adjuvants and contribute significantly to vaccine immunogenicity. Overall, this is a well-designed and executed study, and the results are presented well.

My major concerns are with the significance of these findings, which I think are low for the following reasons: 1) Based on the data presented in Figure S5, the pre-vaccination IFNb levels show a significant association only in aged individuals who received a low dose of the flu vaccine. This is presumably due to lower pre-vaccination antibody levels in this group than in others. No significant correlation was observed between low pre-vaccination IFNb levels and vaccine immunogenicity in young individuals. These results highlight that the adjuvant effects of IFNb are limited to aged individuals who received a low vaccine dose. 2) The adjuvant effects of IL-21 are not new. Previous studies used IL-21 as a vaccine adjuvant, and one NHP study showed adjuvant effects of IL-21 in aged macaques (JCI Insight. 2021;6(20):e150888).

Fig. 6d – Did the authors correct for multiple comparisons for P values? How many times this experiment has been conducted? Ideally, this should be determined longitudinally for each mouse over 6 months, with multiple time points to enhance rigor. Without this, the conclusion regarding the influence of IFNb on antibody persistence is not robust.

Decision Letter:

Our ref: NI-A41802A

19th Mar 2026

Dear Bill,

Thank you for submitting your revised manuscript "Human Vaccine Responses Regulated by Parallel Cytokine Pathways" (NI-A41802A). It has now been seen by the original referees and their comments are below. The reviewers find that the paper has improved in revision, and therefore we'll be happy in principle to publish it in Nature Immunology, pending minor revisions to satisfy the referees' final requests and to comply with our editorial and formatting guidelines.

We will now perform detailed checks on your paper and will send you a checklist detailing our editorial and formatting requirements in about a week. Please do not upload the final materials and make any revisions until you receive this additional information from us.

If you had not uploaded a Word file for the current version of the manuscript, we will need one before beginning the editing

process; please email that to immunology@us.nature.com at your earliest convenience.

Thank you again for your interest in Nature Immunology Please do not hesitate to contact me if you have any questions.

Laurie

Laurie A. Dempsey, Ph.D.
Senior Editor
Nature Immunology
l.dempsey@us.nature.com
ORCID: 0000-0002-3304-796X

Version 1:

Reviewer comments:

Reviewer #2

(Remarks to the Author)
The authors have addressed my concerns satisfactorily.

Open Access This Peer Review File is licensed under a Creative Commons Attribution 4.0 International License, which permits use, sharing, adaptation, distribution and reproduction in any medium or format, as long as you give appropriate credit to the original author(s) and the source, provide a link to the Creative Commons license, and indicate if changes were made.
In cases where reviewers are anonymous, credit should be given to 'Anonymous Referee' and the source.
The images or other third party material in this Peer Review File are included in the article's Creative Commons license, unless indicated otherwise in a credit line to the material. If material is not included in the article's Creative Commons license and your intended use is not permitted by statutory regulation or exceeds the permitted use, you will need to obtain permission directly from the copyright holder.
To view a copy of this license, visit https://creativecommons.org/licenses/by/4.0/

1. Scope and significance of IFN-β as an adjuvant signal

Concern:
Referee #2 notes that the association between pre-vaccination IFN-β levels and vaccine immunogenicity is significant primarily in older adults receiving a low antigen dose, raising concerns about generalizability.

Response and proposed revision:
We agree that the age- and dose-restricted nature of the association should be explicitly framed. We view this restriction not as a limitation, but as biologically and clinically informative. Older adults bear a disproportionate burden of influenza-associated morbidity and mortality and are also the population in which vaccine-induced antibody responses are most consistently suboptimal. Identifying an immune regulator that selectively influences vaccine responsiveness in this high-risk group, therefore, addresses a well-recognized unmet need in human vaccinology.

Mechanistically, our data support a model in which Type I IFN functions as a *conditional adjuvant*, whose impact becomes apparent when antigen availability or innate immune activation is limiting, as is often the case in older individuals receiving standard-dose inactivated vaccines. This interpretation is supported by multiple independent observations in the manuscript, including: (i) the failure of IIV to induce Type I IFN in human immune organoids, (ii) the ability of IFN-β to phenocopy the live-attenuated vaccine cytokine program, and (iii) the disappearance of the IFN-β association when higher antigen doses are used in older adults.

If invited to revise, we would explicitly delimit the scope of this finding in the Results and Discussion, emphasizing that IFN-β regulates vaccine responsiveness under conditions of insufficient innate stimulation rather than acting as a universal adjuvant. These revisions are conceptual and can be implemented immediately.

2. Prior literature on IL-21 as a vaccine adjuvant

Concern:
The adjuvant effects of IL-21 have been reported previously, including in aged non-human primates.

Response and proposed revision:
We fully agree and will clarify this point further. In a revision, we would explicitly position IL-21 not as a novel adjuvant discovery per se, but as a key comparator that helps define a Type I IFN-independent cytokine pathway regulating vaccine responses. The novelty of our study lies in the systematic, side-by-side functional evaluation of multiple cytokines in a human immune organoid system, integrated with human cohort correlates, which allows these pathways to be distinguished quantitatively and mechanistically.

While mouse models have provided important insights into fundamental principles of immune regulation, it remains essential to validate and interrogate immune pathways directly in humans. Numerous cytokines, including IFN-γ[1], IL-18[2], IL-1α/β[2], IL-2[3], and IL-4[3], have been reported to enhance antibody responses to influenza vaccination in diverse murine models. However, in our human spleen organoid system, these cytokines did not enhance vaccine-induced antibody recall (**Fig. 3b**). We will explicitly highlight these distinctions and their translational implications in the revised manuscript.

Importantly, while prior studies have examined IL-21 delivered as recombinant protein or other formats, our in vivo experiments deliver IL-21 via an mRNA–lipid nanoparticle platform. This approach enables localized cytokine expression in draining lymph nodes and directly tests cytokine adjuvanticity in the context of a clinically relevant nucleic-acid delivery modality. We would highlight this distinction more clearly in the revised text and further contextualize our findings within the existing literature.

3. Statistical rigor and durability claims in Figure 6

Concern:
Referee #2 raises issues regarding multiple-comparison correction, experimental repetition, and the robustness of conclusions regarding antibody persistence.

Response and proposed revision:
We agree with these points. In a revision, all relevant P values would be corrected for multiple comparisons (In Figure 6d comparing antibody response durability, the FDR for the comparison between Type I IFN and control is 0.06, while the IL21 and control comparison is 0.78). In addition, we are extending the in vivo studies to include later time points to strengthen the analysis of antibody persistence. The additional antibody measurements and ELISpot assays are already planned and are feasible within 1 month. We also plan to repeat the mouse experiments, but it will take 6 months.

In summary, we believe that the concerns raised by referee #2 can be addressed through focused clarification of scope, a refined framing of novelty, and modest additional experimental support, without altering the study's core conclusions. We would be very grateful for the opportunity to revise the manuscript accordingly and are confident that the proposed revisions are both feasible and timely.

1 van Slooten, M. L. *et al.* Immunoadjuvant activity of interferon-γ-liposomes co-administered with influenza vaccines. *Bba-Mol Cell Biol L* **1531**, 99-110 (2001). https://doi.org/10.1016/S1388-1981(01)00092-0

2 Kayamuro, H. *et al.* Interleukin-1 Family Cytokines as Mucosal Vaccine Adjuvants for Induction of Protective Immunity against Influenza Virus. *J Virol* **84**, 12703-12712 (2010). https://doi.org/10.1128/Jvi.01182-10

3 Herbert, A. S., Heffron, L., Sundick, R. & Roberts, P. C. Incorporation of membrane-bound, mammalian-derived immunomodulatory proteins into influenza whole virus vaccines boosts immunogenicity and protection against lethal challenge. *Virol J* **6** (2009). https://doi.org/10.1186/1743-422X-6-42

Response to Reviewers
Human Vaccine Responses Regulated by Parallel Cytokine Pathways

We thank the editor and both reviewers for their careful, constructive, and insightful evaluation of our manuscript. We are grateful to Reviewer #1 for the positive assessment of the overall approach and significance, and to Reviewer #2 for the thoughtful critiques, which helped us clarify the study's scope, novelty, and rigor. We have revised the manuscript accordingly. Below, we respond point-by-point to Reviewer #2's comments and indicate the precise locations of the revisions by page and line number in the revised manuscript PDF.

Reviewer #2 – Comment 1: Scope and significance of IFN-β as an adjuvant signal

Comment:
Based on the data presented in Figure S5, the pre-vaccination IFNb levels show a significant association only in aged individuals who received a low dose of the flu vaccine. This is presumably due to lower pre-vaccination antibody levels in this group than in others. No significant correlation was observed between low pre-vaccination IFNb levels and vaccine immunogenicity in young individuals. These results highlight that the adjuvant effects of IFNb are limited to aged individuals who received a low vaccine dose.

Response:
We agree that the age- and dose-restricted nature of this association required clearer framing, and we revised the manuscript to explicitly address this point. We now emphasize that this restriction is biologically and clinically informative rather than a limitation, particularly in a population in which vaccine-induced antibody responses are suboptimal. Identifying an immune regulator that selectively influences vaccine responsiveness in this high-risk group, therefore, addresses a well-recognized unmet need in human vaccinology.

Specifically, we clarify that IFN-β functions as a conditional immune regulator whose effects become apparent under conditions of insufficient innate stimulation, when antigen availability or innate immune activation is limiting, a scenario common among older adults receiving standard-dose inactivated vaccines. This interpretation is supported by multiple independent observations in the manuscript, including:

(i) The failure of IIV to induce Type I IFN in human immune organoids.

(ii) The ability of IFN-β to phenocopy the live-attenuated vaccine cytokine program.
(iii) The disappearance of the IFN-β association when higher antigen doses are used in older adults.

Accordingly, we emphasize that IFN-β regulates vaccine responsiveness under conditions of insufficient innate stimulation rather than acting as a universal adjuvant.

We also strengthened the clinical relevance by explicitly noting the disproportionate burden of influenza-associated morbidity, mortality, and poor vaccine responsiveness in this population, addressing a well-recognized unmet need in human vaccinology.

Location of changes (tracked changes only):

- **Results and discussion – IFN-β regulates the human vaccine response among older adults**
  Page 14, lines 11–14
  (Added language framing IFN-β regulation as age-specific and conditional.)
  Page 21, lines 8–10
  (Added text emphasizing the high-risk status of older adults and unmet need for improved adjuvanticity.)

Reviewer #2 – Comment 2: Prior literature on IL-21 as a vaccine adjuvant

Comment:
The adjuvant effects of IL-21 are not new. Previous studies used IL-21 as a vaccine adjuvant, and one NHP study showed adjuvant effects of IL-21 in aged macaques (JCI Insight. 2021;6(20):e150888).

Response:
We agree and revised the manuscript to clarify the role of IL-21. IL-21 is not presented as a novel adjuvant discovery per se. Instead, we explicitly position IL-21 as a comparator cytokine that defines a Type I IFN-independent pathway regulating vaccine responses. While IL-12 has been reported not to induce IL-21 expression in mouse CD4 T cells, it does induce IL-21 in human spleen organoids. The novelty of our study lies in the systematic, side-by-side functional evaluation of multiple cytokines in a human immune organoid system, integrated with human cohort data, enabling quantitative and mechanistic comparison across pathways.

We also strengthened translational framing by explicitly noting that several cytokines reported to enhance antibody responses in murine influenza models do not enhance antibody recall in human spleen organoids, underscoring species-specific differences and the importance of human-relevant functional validation. Numerous cytokines, including IFN-γ, IL-18, IL-1α/β, IL-23, and IL-4, have been reported to enhance antibody

responses to influenza vaccination in diverse murine models; however, in our human spleen organoid system, these cytokines did not enhance vaccine-induced antibody recall. In addition, we clarified that our in vivo studies deliver IL-21 via an mRNA–lipid nanoparticle platform, distinct from prior protein-based approaches. Importantly, while prior studies have examined IL-21 delivered as recombinant protein or other formats, our in vivo experiments deliver IL-21 via an mRNA–lipid nanoparticle platform, enabling localized cytokine expression in draining lymph nodes and directly testing cytokine adjuvanticity in the context of a clinically relevant nucleic-acid delivery modality.

Location of changes (tracked changes only):

- **Abstract**
  Page 3, lines 16–19
  (Added human-specific IL-12→IL-21 induction, defining a Type I IFN-independent pathway.)
- **Results – IL-12 and IL-21 represent a human-specific Type I IFN-independent pathway**
  Page 24, lines 12–17
  (Added explicit statement that IL-12 induces IL-21 in human spleen organoids.)
- **Discussion- mRNA versus protein delivery of cytokines**

  Page 22, lines 20-23

  Cited the work on IL-21 protein adjuvant in mice and highlighted the difference between protein and mRNA delivery
- **Discussion – Human versus mouse divergence**
  Page 24, lines 17–23
  (Added clarification that multiple cytokines reported as adjuvants in mice do not enhance antibody recall in human organoids.)

Reviewer #2 – Comment 3: Statistical rigor and durability claims (Figure 6)

Comment:
Fig. 6d – Did the authors correct for multiple comparisons for P values? How many times this experiment has been conducted? Ideally, this should be determined longitudinally for each mouse over 6 months, with multiple time points to enhance rigor. Without this, the conclusion regarding the influence of IFNb on antibody persistence is not robust.

Response:
We revised the manuscript to strengthen both statistical rigor and mechanistic support

for antibody durability. We extended the longitudinal analysis of the in vivo immunization experiments to 12 months post-boost, demonstrating that the effects of IL-21 and IFN-β mRNA–LNP delivery increase over time. We further added bone-marrow B-cell ELISpot data demonstrating increased formation of antigen-specific long-lived plasma cells, providing direct cellular evidence for sustained humoral immunity. Corresponding figure legends were updated to reflect these analyses. In addition, all relevant P values were correct for multiple comparisons.

Location of changes (tracked changes only):

- **Results – Long-term antibody durability**
  Page 18, lines 2–11
  (Added description of enhanced long-term antibody durability.)
- **Results – Long-lived plasma cell formation**
  Page 19, lines 2–11
  (Added bone-marrow B-cell ELISpot results.)

Summary

In summary, all substantive revisions requested by Reviewer #2 are confined to a small number of clearly delineated tracked changes, primarily within the Results and Discussion sections describing age-specific IFN-β regulation, the IL-12/IL-21 Type I IFN-independent pathway, and the Figure 6 Results and legend detailing 12-month antibody durability. No additional sections of the manuscript were altered beyond these tracked changes. We believe these revisions fully address Reviewer #2’s concerns while preserving the study's central conclusions and translational relevance.
